# Use of Machine Learning to Develop and Evaluate Models Using Preoperative and Intraoperative Data to Identify Risks of Postoperative Complications

**DOI:** 10.1001/jamanetworkopen.2021.2240

**Published:** 2021-03-30

**Authors:** Bing Xue, Dingwen Li, Chenyang Lu, Christopher R. King, Troy Wildes, Michael S. Avidan, Thomas Kannampallil, Joanna Abraham

**Affiliations:** 1 Department of Electrical and Systems Engineering, McKelvey School of Engineering, Washington University in St Louis, St Louis, Missouri; 2 Department of Computer Science and Engineering, McKelvey School of Engineering, Washington University in St Louis, St Louis, Missouri; 3 Institute for Informatics, Washington University in St Louis School of Medicine, St Louis, Missouri; 4 Department of Anesthesiology, Washington University in St Louis School of Medicine, St Louis, Missouri

## Abstract

**Importance:**

Postoperative complications can significantly impact perioperative care management and planning.

**Objectives:**

To assess machine learning (ML) models for predicting postoperative complications using independent and combined preoperative and intraoperative data and their clinically meaningful model-agnostic interpretations.

**Design, Setting, and Participants:**

This retrospective cohort study assessed 111 888 operations performed on adults at a single academic medical center from June 1, 2012, to August 31, 2016, with a mean duration of follow-up based on the length of postoperative hospital stay less than 7 days. Data analysis was performed from February 1 to September 31, 2020.

**Main Outcomes and Measures:**

Outcomes included 5 postoperative complications: acute kidney injury (AKI), delirium, deep vein thrombosis (DVT), pulmonary embolism (PE), and pneumonia. Patient and clinical characteristics available preoperatively, intraoperatively, and a combination of both were used as inputs for 5 candidate ML models: logistic regression, support vector machine, random forest, gradient boosting tree (GBT), and deep neural network (DNN). Model performance was compared using the area under the receiver operating characteristic curve (AUROC). Model interpretations were generated using Shapley Additive Explanations by transforming model features into clinical variables and representing them as patient-specific visualizations.

**Results:**

A total of 111 888 patients (mean [SD] age, 54.4 [16.8] years; 56 915 [50.9%] female; 82 533 [73.8%] White) were included in this study. The best-performing model for each complication combined the preoperative and intraoperative data with the following AUROCs: pneumonia (GBT), 0.905 (95% CI, 0.903-0.907); AKI (GBT), 0.848 (95% CI, 0.846-0.851); DVT (GBT), 0.881 (95% CI, 0.878-0.884); PE (DNN), 0.831 (95% CI, 0.824-0.839); and delirium (GBT), 0.762 (95% CI, 0.759-0.765). Performance of models that used only preoperative data or only intraoperative data was marginally lower than that of models that used combined data. When adding variables with missing data as input, AUROCs increased from 0.588 to 0.905 for pneumonia, 0.579 to 0.848 for AKI, 0.574 to 0.881 for DVT, 0.5 to 0.831 for PE, and 0.6 to 0.762 for delirium. The Shapley Additive Explanations analysis generated model-agnostic interpretation that illustrated significant clinical contributors associated with risks of postoperative complications.

**Conclusions and Relevance:**

The ML models for predicting postoperative complications with model-agnostic interpretation offer opportunities for integrating risk predictions for clinical decision support. Such real-time clinical decision support can mitigate patient risks and help in anticipatory management for perioperative contingency planning.

## Key Points

**Question:**

Can machine learning models predict patient risks of postoperative complications related to pneumonia, acute kidney injury, deep vein thrombosis, delirium, and pulmonary embolism?

**Findings:**

In a cohort study of 111 888 operations at a large academic medical center, machine learning algorithms exhibited high areas under the receiver operating characteristic curve for predicting the risk of postoperative complications related to pneumonia, acute kidney injury, deep vein thrombosis, pulmonary embolism, and delirium.

**Meaning:**

These findings suggest that machine learning models using preoperative and intraoperative data can predict postoperative complications and generate reliable and clinically meaningful interpretations for supporting clinical decisions along the perioperative care continuum.

## Teaser

This cohort study assesses machine learning models for predicting postoperative complications using independent and combined preoperative and intraoperative data and their clinically meaningful model-agnostic interpretations.

## Introduction

More than 10% of surgical patients experience major postoperative complications (eg, myocardial infarction, infection, and blood clots),^1,2,3^ leading to increased mortality, increased need for a higher level of care and management, increased length of postoperative hospital stay, and increased costs of care.^4^ Although some of these postoperative complications are unavoidable because of patient and surgical risk factors,^5^ others are modifiable and potentially preventable through early identification of patient risk factors and administration of evidence-based treatment approaches (eg, timely administration of antibiotics^6,7^ in postoperative care settings).^8,9^

Recent work has highlighted the potential of machine learning (ML) algorithms for predicting postoperative complications. For example, FitzHenry et al^10^ used preoperative patient characteristics and text-based clinical notes to predict 9 major postoperative complications. Others^11,12^ have used a combination of preoperative and a set of descriptive intraoperative features (eg, minimum and maximum of blood pressure values) for similar predictions. In a recent study, Fritz et al^13^ proposed a novel deep learning algorithm that accounted for preoperative and dynamically changing intraoperative data to predict 30-day mortality.

Although these studies^10,11,12,13^ show the potential for using ML algorithms, they have several limitations. First, none of these studies^10,11,12,13^ explicitly separated preoperative and intraoperative data for developing their analytic models. It is therefore difficult to ascertain whether and how preoperative and intraoperative data independently contributed to prediction performance. Second, prior studies^12,13,14^ have acknowledged the high variability of missing data among considered variables and used various standard imputation techniques; however, it is not known how variables with various missing rates can improve prediction performance. This limitation is especially important given that missing data are common during surgery and can have a significant effect on classification accuracy.^15^ Third, and most important, the use of model-agnostic interpretations has been limited^16^; even when such methods have been used, these predictions have relied on statistical features as opposed to clinically meaningful variables.

We focused on 5 postoperative complications in this study: acute kidney injury (AKI), delirium, deep vein thrombosis (DVT), pulmonary embolism (PE), and pneumonia. These 5 complications were selected because they are potentially modifiable during the postoperative period, primarily through early detection and mitigation.^17,18,19,20,21,22,23^ These complications were identified to be relevant and essential for postoperative care management in critical care surgical units based on a recent stakeholder-based study.^24^

In this study, our objectives were 3-fold: (1) to compare the performance of various ML models for predicting postoperative complications using preoperative data, intraoperative data, and combined data for the postoperative complications; (2) to investigate the association of missing input variables with prediction performance; and (3) to develop clinically meaningful, model-agnostic interpretations to support clinical decision-making and care planning.

## Methods

### Setting and Data Sources

Data were obtained from the electronic anesthesia record (MetaVision, iMDSoft) for all adult patients undergoing surgery at a large academic medical center during 4 years (June 1, 2012, to August 31, 2016). Input data elements were extracted from the preoperative assessment record and anesthesia record; the target outcomes related to postoperative complications were retrieved from the patient’s electronic health record.^13^ Data analysis was performed from February 1 to September 31, 2020. The institutional review board of Washington University School of Medicine in St. Louis approved the study with a waiver of consent for this retrospective study. Data were not deidentified. Additional details on study databases and on data extraction and processing are provided in in eAppendix 1 in the Supplement and in the study protocol.^25^ This study used the Transparent Reporting of a Multivariable Prediction Model for Individual Prognosis or Diagnosis (TRIPOD) reporting guideline.

### Outcome Variables

The target outcomes included 5 postoperative complications: AKI, delirium, DVT, PE, and pneumonia. Among these complications, AKI was determined using a combination of laboratory values (serum creatinine) and dialysis event records, and structured anesthesia assessments, laboratory data, and billing data indicating baseline end-stage renal disease were used as exclusion criteria for AKI. Acute kidney injury was defined according to the Kidney Disease Improving Global Outcomes criteria. Delirium was determined from nurse flowsheets (positive Confusion Assessment Method for the Intensive Care unit test result); pneumonia, DVT, and PE were determined based on *International Statistical Classification of Diseases and Related Health Problems, Tenth Revision (ICD-10)* diagnosis codes. Patients without delirium screenings were excluded from the analysis of that complication.

### Data and Data Processing

Input data were split into preoperative and intraoperative variables (Table 1). Preoperative variables included patient characteristics that were available before the surgery, including demographics (eg, age, race, and sex), medical history and acuity (eg, Charlson Comorbidity Index, smoking, and heart failure), physiologic measurements (eg, blood pressure, pulse, and heart rate), and anesthesia type and laboratory measurements (eg, albumin, white blood cells, and glucose). Intraoperative data were time-series variables (captured at 1-minute intervals) and included vital signs (eg, temperature, blood pressure, and heart rate), ventilator settings (eg, tidal volume, inspiratory pressure, and ventilation frequency), and medications (eg, norepinephrine, phenylephrine, and dobutamine). Details on data processing are provided in eAppendix 1 in the Supplement; a description of data types and availability rates of each variable can be found in eAppendix 2 in the Supplement.

**Table 1.** Variables Included in Model(s) and Corresponding Feature Extraction Strategies

Feature type	Features	Preprocessing
Patient characteristics		
Continuous	Age, height, weight, ideal body weight, and BMI	Normalization (*z* score)
Categorical	Sex, race, Charlson Comorbidity Index, functional capacity, ASA physical status, ASA emergency status, anesthesia type, and surgery type	One-hot encoding
Categorical comorbid conditions	Hypertension, coronary artery disease, prior myocardial infarction, congestive heart failure, diastolic function, left ventricular ejection fraction, aortic stenosis, atrial fibrillation, prior stroke or transient ischemic attack, pacemaker or implanted defibrillator, peripheral artery disease, deep venous thrombosis, pulmonary embolism, diabetes, outpatient insulin use, chronic kidney disease, ongoing dialysis, pulmonary hypertension, chronic obstructive pulmonary disease, asthma, obstructive sleep apnea, cirrhosis, any cancer, gastroesophageal reflux, anemia, positive Coombs test result, dementia, and ever-smoker	One-hot encoding if not binary
Continuous preoperative vital signs	Systolic blood pressure, diastolic blood pressure, pulse oximeter, and heart rate	Normalization (*z* score)
Continuous preoperative laboratory values	Albumin, alanine phosphatase, creatinine, glucose, hematocrit, partial thromboplastin time, potassium, sodium, urea nitrogen, and white blood cells	Normalization (*z* score)
Continuous intraoperative vital signs	Systolic blood pressure (invasive and noninvasive), diastolic blood pressure (invasive and noninvasive), mean arterial blood pressure (invasive and noninvasive), central venous pressure, pulse oximeter, temperature, pulse, heart rate, urine output, seconds of electroencephalogram suppression, bispectral index (including spectral edge frequency, electromyographic, total power, and suppression ratio), hematocrit and blood tests of potassium, glucose, base excess, partial pressure of carbon dioxide, blood loss, pH, partial pressure of oxygen, and bicarbonate	Statistical feature extraction (minimum, maximum, mean, entropy, energy, correlation, kurtosis, skewness, and trend) followed by *z* score normalization
Continuous intraoperative ventilatory parameters	Ventilatory frequency, tidal volume, peak inspiratory pressure, positive end-expiratory pressure, fraction of inspired oxygen, end-tidal anesthetic concentration, respiratory minute volume, plateau pressure and expiratory and inspiratory concentration of desflurane, sevoflurane, nitrous oxide, and isoflurane	Statistical feature extraction (minimum, maximum, mean, entropy, energy, correlation, kurtosis, skewness, and trend) followed by *z* score normalization
Continuous intraoperative medications and fluids	Dobutamine, norepinephrine, phenylephrine, epinephrine, and vasopressin	Statistical feature extraction (minimum, maximum, mean, entropy, energy, correlation, kurtosis, skewness, and trend) followed by z score normalization

Abbreviations: ASA, American Society of Anesthesiologists; BMI, body mass index (calculated as weight in kilograms divided by height in meters squared).

### Missing Data

For each preoperative variable, missing data were imputed using the dummy indication technique, where missing fields were replaced by 0s, with indicator vectors representing missingness. For each intraoperative variable, data were imputed using data-level or feature-level imputation. Data-level imputation was applied when a patient had a partially missing time series (ie, the sampling intervals were >1 minute or a data gap in some epochs); in such cases, the series was imputed using the mean value. Feature-level imputation was applied when a patient had a completely missing time series (eg, missing the whole temperature measurements); in such cases, the associated statistical features were categorized as missing and replaced by 0s. Subsequently, a dummy indicator was used to flag the missingness of such time-series variables. Other imputation methods, including fixed-value imputation (mean, median, and mode) and modern imputation methods (missForest,^26^ k nearest neighbor, and multiple imputation by chained equations^27^), were also investigated. Details on these methods are provided in eAppendixes 2 and 3 in the Supplement.

### Feature Engineering

To build a standardized feature space for each of the models, various feature engineering techniques were applied to process the preoperative and intraoperative data (Table 2). One-hot encoding was performed by splitting each categorical variable into binary features in preoperative data, whereas continuous variables were normalized using *z* scores. The processing of intraoperative time series entailed 2 steps: 9 statistical features were computed, including minimum, maximum, mean, entropy, energy, correlation, kurtosis, skewness, and trend. Next, these statistical features were normalized using *z* scores. We extracted 711 features from all clinical variables, including 125 features from preoperative variables, 504 features from intraoperative variables, and 82 unique dummy indicators.

**Table 2.** Characteristics of the Cohort^a^

Characteristic	Finding (N = 111 888)
Age, mean (SD), y	54.4 (16.8)
Female sex	56 914 (50.9)
White race	82 533 (73.8)
Height, median (IQR), cm	170 (163-178)
Weight, median (IQR), kg	83 (69-100)
BMI, median (IQR)	28 (24-34)
Functional capacity, METS	
<4	17 859 (16.0)
4-6	24 978 (22.3)
>6	3094 (3.0)
Missing	64 632 (57.8)
ASA physical status	
1	6828 (6.1)
2	43 758 (39.1)
3	48 809 (43.6)
4	11 858 (10.6)
5	609 (0.5)
ASA emergency status	8544 (7.6)
Surgery type	
Cardiac	3677 (3.3)
Otolaryngology	3186 (2.8)
General	6624 (5.9)
Gynecology	4077 (3.6)
Neurosurgery	3776 (3.4)
Orthopedic	10 416 (9.3)
Thoracic	2568 (2.3)
Urology	4889 (4.4)
Vascular	2669 (2.4)
Others	1825 (1.6)
Unknown	68 181 (60.9)
Hypertension	23 762 (21.2)
Coronary artery disease	7176 (6.4)
Prior myocardial infarction	3582 (3.2)
Congestive heart failure	4198 (3.8)
Atrial fibrillation	2664 (2.4)
Pacemaker or automated implantable cardioverter defibrillator	2061 (1.8)
Prior stroke or transient ischemic attack	1167 (1.0)
Peripheral artery disease	1920 (1.7)
Deep venous thrombosis	3597 (3.2)
Pulmonary embolism	1281 (1.1)
Diabetes mellitus	9331 (8.3)
Outpatient insulin use	7220 (6.5)
Chronic kidney disease	5945 (5.3)
Ongoing dialysis	3938 (3.5)
Pulmonary hypertension	2542 (2.3)
COPD	4311 (3.9)
Asthma	4882 (4.4)
Obstructive sleep apnea	6474 (5.8)
Cirrhosis	585 (0.5)
Any cancer	12 211 (10.9)
Gastroesophageal reflux	15 543 (13.9)
Anemia	12 333 (11.0)
Positive Coombs test result	683 (0.6)
Dementia	316 (0.3)
Ever-smoker	63 797 (57.0)

Abbreviations: ASA, American Society of Anesthesiologists; BMI, body mass index (calculated as weight in kilograms divided by height in meters squared); COPD, chronic obstructive pulmonary disease; IQR, interquartile range; METS, metabolic equivalents.

^a^ Data are expressed as number (percentage) of participants unless otherwise indicated.

### ML Models

Both linear and nonlinear ML models were applied to the 3 data sets: the preoperative, intraoperative, and combined data sets. Linear models included support vector machine and logistic regression, and nonlinear models included random forest, gradient boosting tree (GBT), and deep neural network (DNN). The support vector machine, logistic regression, and random forest models were implemented using the Python Sklearn package.^28^ The GBT was implemented using the Xgboost package,^29^ and DNN was implemented using TensorFlow.^30^ Code and configurations of ML models are provided in eAppendixes 3 and 4 in the Supplement.

### Model Performance and Evaluation

**Figure 1.**
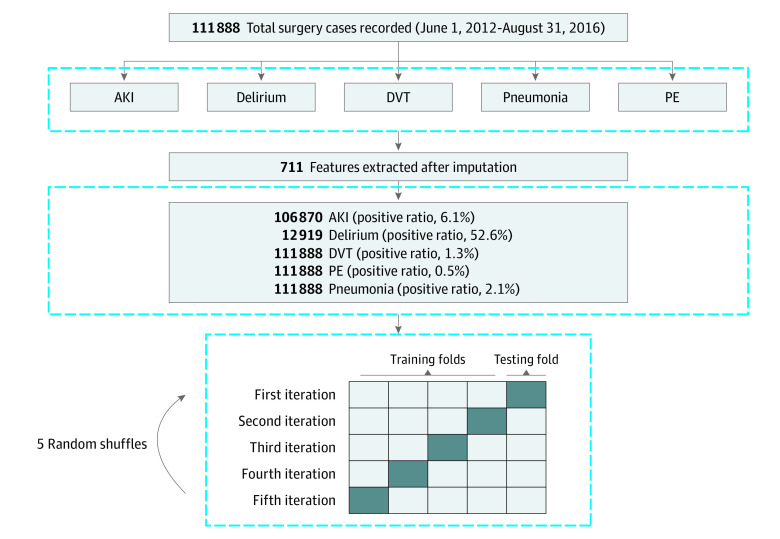
Flowchart of Complication Analysis and Cohort Split AKI indicates acute kidney injury, DVT, deep vein thrombosis; PE, pulmonary embolism.

Because of the rare occurrence of certain complications (positive ratio <2% for DVT and <1% for PE) (Figure 1), the model performance obtained from a random split of training and testing data may not be generalizable. To develop an unbiased assessment of model performance, we performed 5 random shuffles of 5-fold cross-validation. Each iteration used a different stratified fold for model evaluation, and the remaining folds were used for model training. At the training stage, rare events were up-sampled, based on the positive event rate of each complication, with random replacement using the Imblearn package,^31^ to produce a training data set with balanced positive and negative labels.

Seven performance measures were recorded in each iteration, including the area under the receiver operating characteristic curve (AUROC), the area under the precision recall curve, accuracy, sensitivity, specificity, precision, and F scores. Models were compared in each data set using the mean AUROCs from 5 × 5 iterations. For each complication, the best-performing ML model (measured by its AUROC) was chosen.

### Model Interpretation

We used the Shapley Additive Explanations (SHAP)^32^ for interpreting model predictions. SHAP is a model-agnostic explanation technique that helps in interpreting the results from a predictive model. The interpretation was based on the SHAP value for each feature, representing the contribution of a feature to the predicted risk of a complication. A positive SHAP value indicated that the corresponding feature contributes to a higher risk of the complication, whereas a negative SHAP value indicated that the corresponding feature leads to a lower risk of that complication. The magnitude of SHA*P* values represented the contribution of that feature toward prediction performance.

Because ML features in models are not clinically meaningful, we transformed the SHA*P* values from the ML feature space to the corresponding clinical variable space, so that every transformed SHAP value mapped back to an original preoperative or intraoperative variable. For all categorical variables, the SHA*P* values were calculated as the sum of the SHA*P* values of its one-hot encoded features. For intraoperative time-series variables, the SHA*P* values were calculated as the sum of the SHA*P* values of each of its statistical features. When variables had missing data, the SHA*P* values included the contribution of each of the dummy indicators.

We developed a pragmatic visualization for model interpretation at the patient level. This visualization compared a patient (ie, any selected patient) with the cohort of patients who did not experience the selected complication. The top 10 variables with highest SHA*P* values (ie, corresponding to the most significant influence on the prediction) associated with the selected patient were sorted and included to highlight key features. We depicted the following to support practitioners’ model interpretation: (1) the accumulated risk with the each of the top 10 clinical variables, measured by SHA*P* values and scaled to on a 0- to 1-point scale, with 0 indicating lowest risk score and 1 indicating highest risk score, using a logistic function; (2) comparison of the risk contributions of each of these top 10 clinical variables of the selected patient to the risk contributions of the average patient not in that complication cohort; and (3) characterization of significant intraoperative time series in terms of its statistical features.

### Statistical Analysis

Two different analyses were conducted. In the first analysis, we used 3 data sets: preoperative data, intraoperative data, and the combination of both data sets. We used the features in each data set for model training and testing. In the second analysis, we evaluated improvements in predictive performance by incorporating variables with various missing rates. All features were sorted in ascending order of their missing rates. We started using features that were available for all patients (ie, complete case analysis with overall missing rate of 0%); then we added more features (ie, ones with missing data) and recorded the predictive performance with respect to the overall missing rates and the number of features.

## Results

 A total of 111 888 patients (mean [SD] age, 54.4 [16.8] years; 56 915 [50.9%] female; 82 533 [73.8%] White) were included in this study. (Table 2). The mean duration of follow-up was based on postoperative length of stay (mean [SD], 11.138 days). The resulting data sets contained 106 870 patients with AKI (positive event rate, 6.1%), 12 919 with delirium (positive event rate, 52.6%), 111 888 with DVT (positive event rate, 1.3%), 111 888 with PE (positive event rate, 0.5%), and 111 888 with pneumonia (positive event rate, 2.1%) (Figure 1). The positive event rates were held consistent in each train-test split.

### Model Performance

**Figure 2.**
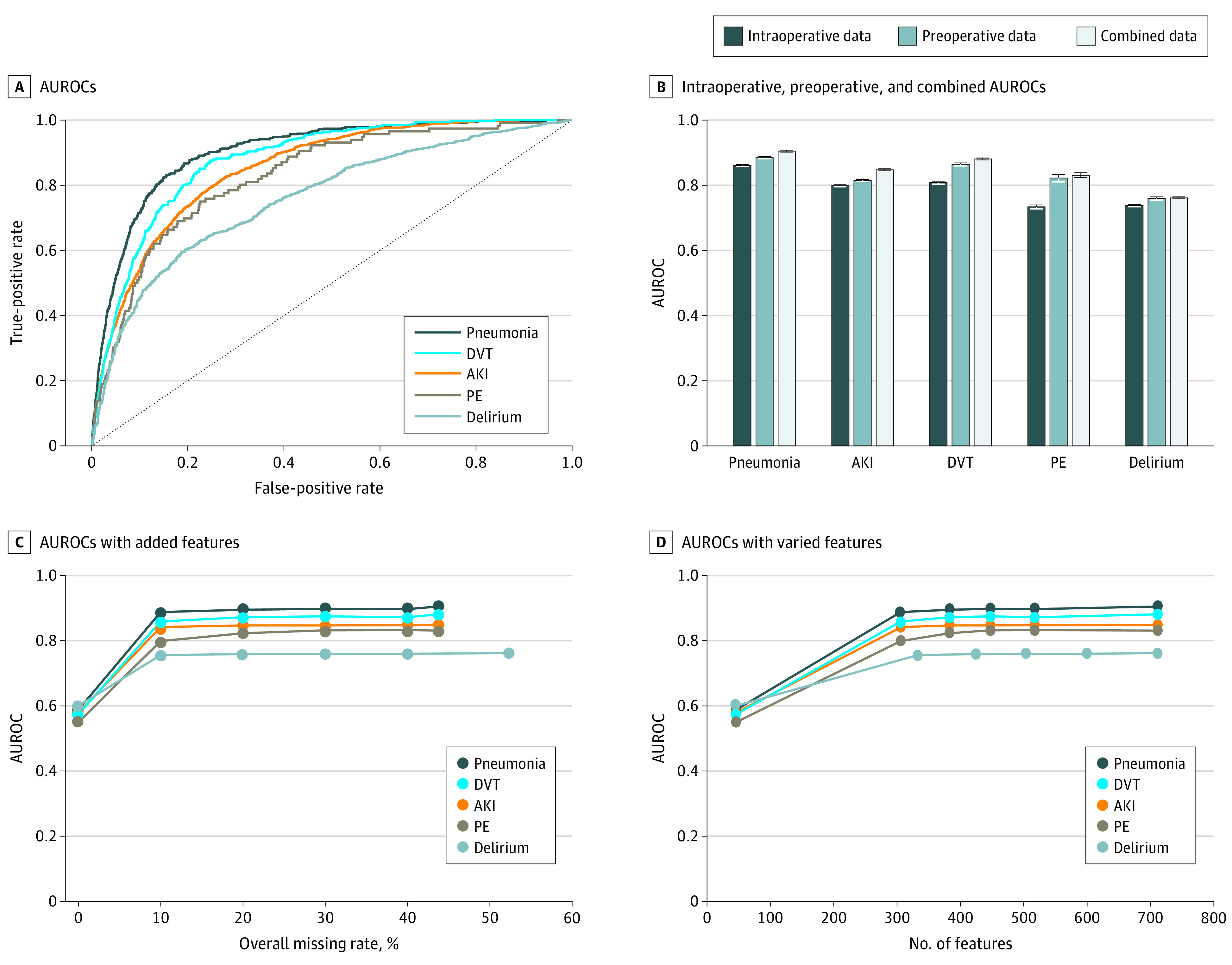
Results of Machine Learning Models A, Areas under the receiver operating characteristic curve (AUROCs) of best-performing learning models. B, AUROCs when using only preoperative data, intraoperative data, and combined data. C, AUROCs with added features in ascending order of missing rate. D, AUROCs with varied number of features. AKI indicates acute kidney injury; DVT, deep vein thrombosis; PE, pulmonary embolism. The error bars indicate 95% CIs.

Of the considered ML models, the best-performing models were GBT for pneumonia, AKI, DVT, and delirium and DNN for PE. The AUROCs for these models were as follows: 0.905 (95% CI, 0.903-0.907) for pneumonia, 0.848 (95% CI, 0.846-0.851) for AKI, 0.881 (95% CI, 0.878-0.884) for DVT, 0.831 (95% CI, 0.824-0.839) for PE, and 0.762 (95% CI, 0.759-0.765) for delirium (Figure 2A; see eAppendix 5 in the Supplement for detailed performance metrics, including area under the precision recall curve, accuracy, sensitivity, specificity, F score, and precision). We further compared the prediction performance of various imputation methods on the pneumonia data set and found that the dummy indication technique achieved the best performance (see eAppendix 3 in the Supplement for detailed comparisons).

Across all complications, the predictive performance using only the preoperative data set was better than using only the intraoperative data set; the combined data set had the best performance for all complications. However, models with only the preoperative data set performed nearly as well. The difference in AUROC between the combined and preoperative-only data sets were 0.019 for pneumonia, 0.032 for AKI, 0.016 for DVT, 0.009 for PE, and 0.002 for delirium (Figure 2B).

When adding features with greater missing rates, there was a consistent increase in the AUROC: 0.588 to 0.905 for pneumonia, 0.579 to 0.848 for AKI, 0.574 to 0.881 for DVT, and 0.6 to 0.762 for delirium (Figure 2C). The predictive performance for all outcomes flattened when the number of features was greater than 400 (Figure 2D).

### Model Interpretation

To highlight the clinical utility and translational impact of such predictions in perioperative care, we present a case example of a patient with a positive predicted risk for pneumonia.

A 65-year-old patient with fever, a history of chronic obstructive pulmonary disease, heavy smoking, and elevated liver enzymes is admitted for an open pneumonectomy. An epidural is placed preoperatively. The patient is given a moderate dose of phenylephrine intraoperatively (maximum dose, 0.8 μg/kg per minute) and 2.5 L of crystalloid fluids, and a right chest tube is placed. The patient is extubated in the operating room and transferred to the intensive care unit with a high-flow face mask (9 L of oxygen).

**Figure 3.**
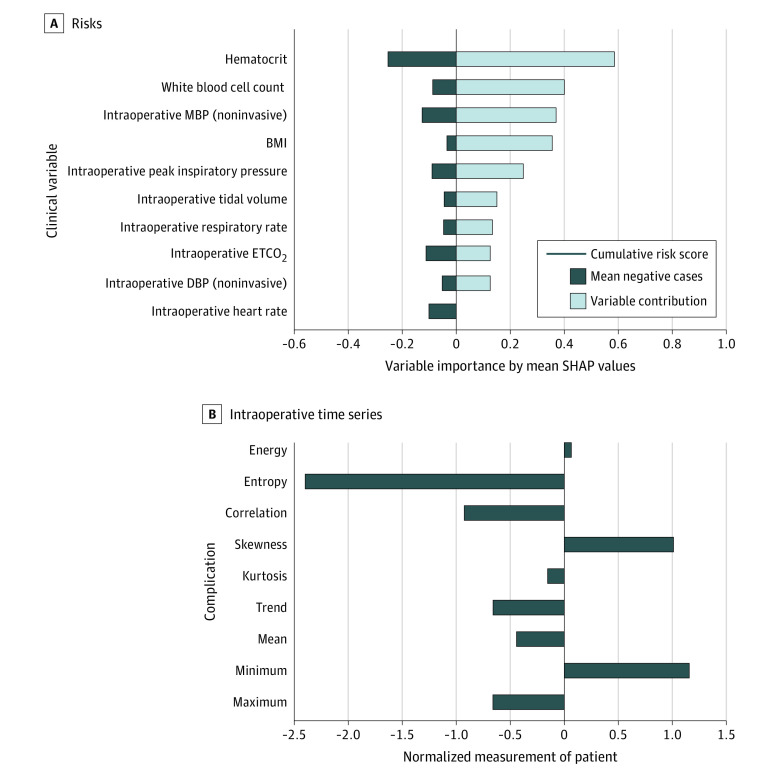
Complication-Specific Model Interpretation A, Evolvement of risks (from top to bottom) contributed by each variable (magnitude of contribution decreasing from left to right) compared with a group of patients who did not have pneumonia. B, Characterization of significant intraoperative time series (in this case, it is the noninvasive mean blood pressure [MBP]) by its statistical features. Each statistical feature is normalized to zero mean and unit variance; therefore, the magnitude reflects its deviation from the historical mean of patients. BMI indicates body mass index (calculated as weight in kilograms divided by height in meters squared); ETCO_2_, end-tidal carbon dioxide; SHAP, Shapley Additive Explanations.

A patient undergoing pneumonectomy is at high risk for pulmonary complications,^33^ including pneumonia. For this patient, the ML model predicted the patient to be at risk for pneumonia. Using the best-performing GBT model (cross-validated AUROC, 0.905, overall accuracy on validation data set, 94.1%), we illustrate the complication-specific interpretation, depicting the risk contributors to pneumonia. As shown in Figure 3, the key contributors to the model prediction were the patient’s anemia (hematocrit) and low body mass index, attributed to their chronic condition; elevated white blood cell count, a possible reflection of baseline infection; and tidal volume and respiratory rate signals, a potential reflection of the transition to single-lung ventilation.

Although these features are not meant to be necessarily causal or modifiable, in this example, the ML output explanations highlight the relevant features associated with pneumonia. These insights after surgery can inform appropriate clinical actions in the intensive care unit, including early mobilization, pulmonary hygiene with a respiratory therapist (eg, incentive spirometry), scheduled bronchodilators, continuing epidural analgesia, supplemental oxygen, close monitoring, and a low threshold for antibiotic therapy. When compared with a cohort of patients that did not develop pneumonia, 9 of the 10 clinical variables with the highest SHA*P* values (ie, variables that contributed most to the risk) classified the patient to be at risk for pneumonia. The addition of these top 10 clinical variables increases the overall risk of getting pneumonia from 0.500 to 0.920 (calculated by the scaled SHA*P* values).

Additional types of visualizations are provided in eAppendix 6 in the Supplement. Model interpretation in the cases of false-positive, false-negative, true-positive, and true-negative predictions are presented for each outcome in eAppendix 7 in the Supplement.

## Discussion

This cohort study used a ML approach with preoperative and intraoperative surgical data, both independently and in combination, to predict the occurrence of postoperative surgical complications. Gradient boosting tree achieved the best predictive performance for pneumonia, AKI, DVT, and delirium, and DNN had the best predictive performance for PE. This superior performance of GBT and DNN is indicative of the complexity of input space, where simple linear algorithms (eg, logistic regression and support vector machine) were not able to capture important patterns for prediction.

Prior studies^11,13,34,35^ have used the available data en masse without accounting for the time of data availability in the perioperative continuum. For example, certain clinical variables are available before the surgery, including laboratory results, demographic characteristics, and patient clinical characteristics. Characterizing the time of availability of specific clinical data elements can help make predictions about the patient’s potential clinical trajectory. For ascertaining the predictive capabilities at the preoperative phase and the immediate postoperative phase, separate models that used preoperative, intraoperative, and combined data sets were developed.

Given that the predictive performance of the models using the combined data set was only marginally better than those with only the preoperative data, there is potential utility of these models in multiple surgical scenarios. For example, these models can be generated for preoperative predictions (using data available before surgery) and postoperative complication predictions (either with the combined data set when available or with only intraoperative data for off-hour unplanned patient operations without preoperative data). Practitioners can use these predictions to develop perioperative care management goals and care plans. For example, practitioners can highlight the postoperative risks for patient complications during handoff communication between the operating room and a critical care unit, which can help formulate a contingency plan based on identified risks and the associated factors identified from the model interpretations.

Previous studies^12,13,14^ have not explicitly addressed the effect of missing values on predictive performance. In the present analysis, missing variables were systematically included in the modeling approach to evaluate the associations of missingness with predictive performance. The results of this study demonstrated that the inclusion of missing variables improves prediction performance; however, the performance improvement reached an asymptote for all complications with a large number of features.

This study explored a model-agnostic interpretation technique for describing potential clinical factors that contribute to postoperative complications. Although the model interpretation techniques developed in the ML community were primarily targeted at data scientists, this study extended the interpretation techniques to facilitating meaningful use in clinical communication, such as for patient handoff communication. As opposed to estimating the contributions of features extracted from the original clinical data, this study used a systematic approach that maps the features extracted from both preoperative and intraoperative variables back to the clinical variable space to generate clinically meaningful interpretation. Leveraging SHAP-based analysis, this study generated a visualization format for interpreting patient-associated risks based on the clinical variables. By highlighting significant clinical variables (ie, interpretations) that contribute to the risk predictions, such visualization can assist practitioners in preemptive and early identification of key factors, including modifiable ones, that contribute to patients’ risk of developing a complication. Practitioners can use such insights to quickly identify potential factors that contribute to a complication risk and decide the evidence-based treatment protocols to mitigate such risks.

Furthermore, as highlighted by the case example, the prediction algorithm can be valuable in validating or assisting practitioners in ascertaining the risk of postoperative complications, highlighting additional clinical nuances that may explain these risks (which may have been previously omitted), and providing cognitive support to augment postoperative proximal practitioner decisions.

### Limitations

This study has limitations. First, the surgical patient data were obtained from a single hospital. Second, several variables were not accounted for in the models, including planned surgical description, length of surgery, key intraoperative variables (eg, blood transfusion data, and urine output), and commonly used vasopressors, inotropes, and certain medications used during surgery specified by consultants,^13^ which potentially could affect the model performance. Third, subgroup analysis based on the various surgery types was not conducted because of the small number of patients within each subgroup; hence, the clinical utility of the predictions of postoperative complications based on specific surgery types is limited. Fourth, the target outcomes (except AKI) were identified using administrative data (eg, *ICD-10*–based discharge diagnosis codes) and were not verified using manual health record reviews. The validity of the outcomes determined by automated health record review has previously been compared with manual record review and patient-reported outcomes.^36^ Similar to another report,^37^ this previous study^36^ found large positive likelihood ratios with moderate sensitivity. Another study^38^ found that practitioners and coders have substantial disagreement, largely around the severity of a complication. Others^39,40^ have found medium to high sensitivity (70%) for *ICD-10*–based detection of in-hospital pneumonia and DVT. To address this limitation, the current ongoing work involves data triangulation across the administrative data, clinical text, and other data to align with high-quality manual health record review provided by National Surgical Quality Improvement Program adjudicators. Fifth, state-of-the-art model interpretation approaches, including SHAP and its alternatives,^32,41,42^ do not consider the dependencies between features and inevitably introduce a correlation bias.^32,42,43^

## Conclusions

These findings suggest that the proposed ML framework for predicting postoperative complications with model-agnostic interpretation affords opportunities for implementing and integrating ML output in real-time clinical decision support systems and anticipatory management tools for practitioners to support their postoperative care planning and resource management.

## Notes

Supplement. **eAppendix 1.** Data Extraction Steps **eAppendix 2.** List of Variables and Data Type **eAppendix 3.** Exploration on Data Imputation **eAppendix 4.** Model Development **eAppendix 5.** Details of Performance Metrics of Each Model **eAppendix 6.** More on Model Interpretation **eAppendix 7.** Model Interpretation on False Positives, False Negatives, True Positives, and True Negatives **eAppendix 8.** Three Scenarios for Predicting Postoperative Complications

## References

[1] Hamel MB, Henderson WG, Khuri SF, Daley J. Surgical outcomes for patients aged 80 and older: morbidity and mortality from major noncardiac surgery. J Am Geriatr Soc. 2005;53(3):424-429. doi:10.1111/j.1532-5415.2005.53159.x 15743284

[2] Turrentine FE, Wang H, Simpson VB, Jones RS. Surgical risk factors, morbidity, and mortality in elderly patients. J Am Coll Surg. 2006;203(6):865-877. doi:10.1016/j.jamcollsurg.2006.08.026 17116555

[3] Healey MA, Shackford SR, Osler TM, Rogers FB, Burns E. Complications in surgical patients. Arch Surg. 2002;137(5):611-617. doi:10.1001/archsurg.137.5.611 11982478

[4] Tevis SE, Kennedy GD. Postoperative complications and implications on patient-centered outcomes. J Surg Res. 2013;181(1):106-113. doi:10.1016/j.jss.2013.01.032 23465392 PMC3637983

[5] Hollinger A, Siegemund M, Goettel N, Steiner LA. Postoperative delirium in cardiac surgery: an unavoidable menace? J Cardiothorac Vasc Anesth. 2015;29(6):1677-1687. doi:10.1053/j.jvca.2014.08.021 26456271

[6] Young PY, Khadaroo RG. Surgical site infections. Surg Clin North Am. 2014;94(6):1245-1264. doi:10.1016/j.suc.2014.08.008 25440122

[7] Bratzler DW, Houck PM; Surgical Infection Prevention Guideline Writers Workgroup. Antimicrobial prophylaxis for surgery: an advisory statement from the National Surgical Infection Prevention Project. Am J Surg. 2005;189(4):395-404. doi:10.1016/j.amjsurg.2005.01.015 15820449

[8] Kable AK, Gibberd RW, Spigelman AD. Adverse events in surgical patients in Australia. Int J Qual Health Care. 2002;14(4):269-276. doi:10.1093/intqhc/14.4.269 12201185

[9] Gawande AA, Thomas EJ, Zinner MJ, Brennan TA. The incidence and nature of surgical adverse events in Colorado and Utah in 1992. Surgery. 1999;126(1):66-75. doi:10.1067/msy.1999.98664 10418594

[10] FitzHenry F, Murff HJ, Matheny ME, . Exploring the frontier of electronic health record surveillance: the case of postoperative complications. Med Care. 2013;51(6):509-516. doi:10.1097/MLR.0b013e31828d1210 23673394 PMC3658153

[11] Hofer IS, Lee C, Gabel E, Baldi P, Cannesson M. Development and validation of a deep neural network model to predict postoperative mortality, acute kidney injury, and reintubation using a single feature set. NPJ Digit Med. 2020;3(1):58. doi:10.1038/s41746-020-0248-0 32352036 PMC7170922

[12] Weller GB, Lovely J, Larson DW, Earnshaw BA, Huebner M. Leveraging electronic health records for predictive modeling of post-surgical complications. Stat Methods Med Res. 2018;27(11):3271-3285. doi:10.1177/0962280217696115 29298612

[13] Fritz BA, Cui Z, Zhang M, . Deep-learning model for predicting 30-day postoperative mortality. Br J Anaesth. 2019;123(5):688-695. doi:10.1016/j.bja.2019.07.025 31558311 PMC6993109

[14] Warner JL, Zhang P, Liu J, Alterovitz G. Classification of hospital acquired complications using temporal clinical information from a large electronic health record. J Biomed Inform. 2016;59:209-217. doi:10.1016/j.jbi.2015.12.008 26707449 PMC4792687

[15] Wang LE, Shaw PA, Mathelier HM, Kimmel SE, French B. Evaluating risk-prediction models using data from electronic health records. Ann Appl Stat. 2016;10(1):286-304. doi:10.1214/15-AOAS891 27158296 PMC4859766

[16] Lundberg SM, Nair B, Vavilala MS, . Explainable machine-learning predictions for the prevention of hypoxaemia during surgery. Nat Biomed Eng. 2018;2(10):749-760. doi:10.1038/s41551-018-0304-0 31001455 PMC6467492

[17] Meersch M, Schmidt C, Hoffmeier A, . Prevention of cardiac surgery-associated AKI by implementing the KDIGO guidelines in high risk patients identified by biomarkers: the PrevAKI randomized controlled trial. Intensive Care Med. 2017;43(11):1551-1561. doi:10.1007/s00134-016-4670-3 28110412 PMC5633630

[18] Cavallazzi R, Saad M, Marik PE. Delirium in the ICU: an overview. Ann Intensive Care. 2012;2(1):49. doi:10.1186/2110-5820-2-49 23270646 PMC3539890

[19] Janssen TL, Alberts AR, Hooft L, Mattace-Raso F, Mosk CA, van der Laan L. Prevention of postoperative delirium in elderly patients planned for elective surgery: systematic review and meta-analysis. Clin Interv Aging. 2019;14:1095-1117. doi:10.2147/CIA.S201323 31354253 PMC6590846

[20] Vlisides P, Avidan M. Recent advances in preventing and managing postoperative delirium. *F1000Res*. 2019;8. doi:10.12688/f1000research.16780.1 PMC6498743 31105934

[21] Caparelli ML, Shikhman A, Jalal A, Oppelt S, Ogg C, Allamaneni S. prevention of postoperative pneumonia in noncardiac surgical patients: a prospective study using the National Surgical Quality Improvement Program database. Am Surg. 2019;85(1):8-14. doi:10.1177/000313481908500104 30760338

[22] Miskovic A, Lumb AB. Postoperative pulmonary complications. Br J Anaesth. 2017;118(3):317-334. doi:10.1093/bja/aex002 28186222

[23] Cayley WE Jr. Preventing deep vein thrombosis in hospital inpatients. BMJ. 2007;335(7611):147-151. doi:10.1136/bmj.39247.542477.AE 17641348 PMC1925160

[24] Abraham J, King CR, Meng A. Ascertaining design requirements for postoperative care transition interventions. *Appl Clin Inform.* 2021;12(1):107-115. doi:10.1055/s-0040-1721780 PMC7904383 33626584

[25] Fritz BA, Chen Y, Murray-Torres TM, . Using machine learning techniques to develop forecasting algorithms for postoperative complications: protocol for a retrospective study. BMJ Open. 2018;8(4):e020124. doi:10.1136/bmjopen-2017-020124 29643160 PMC5898287

[26] Stekhoven DJ, Bühlmann P. MissForest—non-parametric missing value imputation for mixed-type data. Bioinformatics. 2012;28(1):112-118. doi:10.1093/bioinformatics/btr597 22039212

[27] Azur MJ, Stuart EA, Frangakis C, Leaf PJ. Multiple imputation by chained equations: what is it and how does it work? Int J Methods Psychiatr Res. 2011;20(1):40-49. doi:10.1002/mpr.329 21499542 PMC3074241

[28] Pedregosa F, Varoquaux G, Gramfort A, . Scikit-learn: machine learning in Python. J Machine Learn Res. 2011;12(85):2825-2830.

[29] Chen T, Guestrin C. XGBoost: a scalable tree boosting system. In: *Proceedings of the 22nd ACM SIGKDD International Conference on Knowledge Discovery and Data Mining (KDD ‘16)*. Association for Computing Machinery; 2016:785-794. doi:10.1145/2939672.2939785

[30] Abadi M, Agarwal A, Barham P, . TensorFlow: large-scale machine learning on heterogeneous distributed systems. *arXiv.* Preprint posted online March 14, 2016.

[31] Lemaître G, Nogueira F, Aridas CK. Imbalanced-learn: A Python toolbox to tackle the curse of imbalanced datasets in machine learning. J Machine Learn Res. 2017;18(17):1-5.

[32] Lundberg S, Lee S-I. A unified approach to interpreting model predictions. *arXiv.* Preprint posted online May 22, 2017.

[33] Liu G-W, Sui X-Z, Wang S-D, Zhao H, Wang J. Identifying patients at higher risk of pneumonia after lung resection. J Thorac Dis. 2017;9(5):1289-1294. doi:10.21037/jtd.2017.04.42 28616280 PMC5465142

[34] Meyer A, Zverinski D, Pfahringer B, . Machine learning for real-time prediction of complications in critical care: a retrospective study. Lancet Respir Med. 2018;6(12):905-914. doi:10.1016/S2213-2600(18)30300-X 30274956

[35] Brajer N, Cozzi B, Gao M, . Prospective and external evaluation of a machine learning model to predict in-hospital mortality of adults at time of admission. JAMA Netw Open. 2020;3(2):e1920733-e1920733. doi:10.1001/jamanetworkopen.2019.20733 32031645 PMC12068827

[36] Fritz BA, Escallier KE, Ben Abdallah A, . Convergent validity of three methods for measuring postoperative complications. Anesthesiology. 2016;124(6):1265-1276. doi:10.1097/ALN.0000000000001108 27028469 PMC5083125

[37] Kaafarani HMA, Rosen AK. Using administrative data to identify surgical adverse events: an introduction to the Patient Safety Indicators. Am J Surg. 2009;198(5)(suppl):S63-S68. doi:10.1016/j.amjsurg.2009.08.008 19874937

[38] Henry LR, Minarich MJ, Griffin R, . Physician derived versus administrative data in identifying surgical complications: fact versus fiction. Am J Surg. 2019;217(3):447-451. doi:10.1016/j.amjsurg.2018.08.015 30180936

[39] Alotaibi GS, Wu C, Senthilselvan A, McMurtry MS. The validity of *ICD* codes coupled with imaging procedure codes for identifying acute venous thromboembolism using administrative data. Vasc Med. 2015;20(4):364-368. doi:10.1177/1358863X15573839 25834115

[40] Higgins TL, Deshpande A, Zilberberg MD, . Assessment of the accuracy of using *ICD-9* diagnosis codes to identify pneumonia etiology in patients hospitalized with pneumonia. JAMA Netw Open. 2020;3(7):e207750-e207750. doi:10.1001/jamanetworkopen.2020.7750 32697323 PMC7376393

[41] Schwab P, Karlen W. CXPlain: causal explanations for model interpretation under uncertainty. In: Wallach H, Larochelle H, Beygelzimer A, Garnett R, eds. *Advances in Neural Information Processing Systems.* Vol 32. Curran Associates; 2019:10220-10230.

[42] Ribeiro MT, Singh S, Guestrin C. “Why should I trust you?”: explaining the predictions of any classifier. *arXiv.* Preprint posted online August 9, 2016.

[43] Aas K, Jullum M, Løland A. Explaining individual predictions when features are dependent: more accurate approximations to Shapley values. *arXiv*. Preprint posted online February 6, 2020.

