# Supplementary material for: Use of Machine Learning to Develop and Evaluate Models Using Preoperative and Intraoperative Data to Identify Risks of Postoperative Complications
Source: JAMA Netw Open. 2021 Mar 30;4(3):e212240. doi: 10.1001/jamanetworkopen.2021.2240 (PMC8010590; doi:10.1001/jamanetworkopen.2021.2240)
Supplement: Supplement. — eAppendix 1. Data Extraction Steps eAppendix 2. List of Variables and Data Type eAppendix 3. Exploration on Data Imputation eAppendix 4. Model Development eAppendix 5. Details of Performance Metrics of Each Model eAppendix 6. More on Model Interpretation eAppendix 7. Model Interpretation on False Positives, False Negatives, True Positives, and True Negatives eAppendix 8. Three Scenarios for Predicting Postoperative Complications [file jamanetwopen-e212240-s001.pdf]

Supplementary Online Content

Xue B, Li D, Lu C, et al. Use of machine learning to develop and evaluate models using preoperative and intraoperative data to identify risks of postoperative complications. *JAMA Netw Open*. 2021;4(3):e212240. doi:10.1001/jamanetworkopen.2021.2240

eAppendix 1. Data Extraction Steps

eAppendix 2. List of Variables and Data Type

eAppendix 3. Exploration on Data Imputation

eAppendix 4. Model Development

eAppendix 5. Details of Performance Metrics of Each Model

eAppendix 6. More on Model Interpretation

eAppendix 7. Model Interpretation on False Positives, False Negatives, True Positives, and True Negatives

eAppendix 8. Three Scenarios for Predicting Postoperative Complications

This supplementary material has been provided by the authors to give readers additional information about their work.

© 2021 Xue B et al. *JAMA Network Open.*

eAppendix 1. Data Extraction Steps

Methods

In this appendix, we first describe the following major tasks that we have accomplished for data extraction:

(1) Use filters to clean the data
(2) Capture various preoperative laboratory values
(3) Create postoperative outcomes

These tasks are outlined sequentially on the following pages. For other outcomes (pneumonia, DVT, PE and delirium), refer to "**SATISFY-SOS Pilot Study – Algorithm for Automated Medical Record Review"** at the end of this appendix.

TASK 1: Use filters to clean the data

- In the laboratory files (including the creatinine files, preoperative labs, and in-hospital labs)
  - Eliminate rows where NORMALIZED_RESULT is a text value rather than a number.
  - Eliminate rows with excluded LAB_CODE based on eTable 1. (This is primarily to exclude tests being run on fluids other than blood.)
  -

eTable 1. Lab Codes to Include and Exclude for Laboratory Values

Laboratory Value	LAB CODE to Include	LAB CODE to Exclude
Alanine Transaminase (ALT)	445072	
Albumin	132	3620<br>141104<br>158014
Alkaline Phosphatase	445038	
Bicarbonate	4126<br>5297	
Creatinine	695<br>141732	6759
Glucose	1283<br>1585<br>7384<br>18614<br>2300<br>1484<br>40290	866<br>1681<br>4345<br>4821<br>7385
Hematocrit	1800<br>32512	5569<br>20867
Partial Thromboplastin Time (PTT)	1142<br>8988	
Potassium	6424<br>6426<br>7848	1265<br>9018

© 2021 Xue B et al. *JAMA Network Open.*

Sodium	1456<br>8765	7026
Urea Nitrogen (BUN)	1456<br>8765	7026
White Blood Cells (WBC)	3554<br>5148	8183<br>496026

TASK 2: Capture preoperative laboratory values

- For each lab test listed in Table 1, identify the value that is closest to the anesthesia start time, but is still before the anesthesia start time.
  - Consider all similarly-named variables as a group. (For example, the preoperative bicarbonate would be the value closest to anesthesia start, regardless of whether it is lab code 4126 or 5297)
  - Include labs drawn up to 30 days before the anesthesia start time.
  - If a patient did not have a particular lab drawn, then treat that field as missing.

Thus each patient should have variables called "pre op ALT," "pre op albumin," and so on.

TASK 3: Create two variables for postoperative complications: Acute kidney injury (AKI)

The first variable uses creatinine values to define AKI. A patient has an AKI if the creatinine rises by 0.3 mg/dl or by 50% of its preoperative value within the first 48 hours after surgery.

The second variable uses new onset of renal replacement therapy (dialysis) to define AKI. A patient has an AKI if they were not on dialysis before surgery and they needed dialysis after surgery prior to discharge.

Patients are excluded if they are undergoing kidney transplant or if they are undergoing creation or revision of dialysis access (for example, arteriovenous fistula, ateriovenous graft). eTable 2 at the end of this section contains CPT codes for these procedures, eTable 3 contains ICD-9-CM codes, and Table 4 contains ICD-10-PCS codes.

(1) Acute Kidney Injury – Creatinine Definition

This calculation requires the CKD_DialysisHistory variable.

- Set the AKI_Creatinine variable to missing if any of the following are true
  - CKD_DialysisHistory = "ongoing hemodialysis" or "ongoing peritoneal dialysis"
  - Preoperative creatinine is missing
  - Postoperative creatinine peak is missing
  - Procedure code matches a CPT code in Table 2, ICD-9-CM code in Table 3, or ICD-10-PCS code in Table 4. (In other words, patient is undergoing kidney transplant or dialysis access procedure)
- Set the AKI_Creatinine variable to 1 if any of the following are true
  - Postoperative peak creatinine >= Preoperative creatinine + 0.3
  - Postoperative peak creatinine >= 1.5 * Preoperative creatinine
- Otherwise set the AKI_Creatinine variable to 0

(2) Acute Kidney Injury – Dialysis Definition

© 2021 Xue B et al. *JAMA Network Open.*

This calculation requires the M_Kidney_Dialysis variable and the CKD_DialysisHistory variable.

- Set the AKI_Dialysis variable to missing if any of the following are true
  - CKD_DialysisHistory = "ongoing hemodialysis" or "ongoing peritoneal dialysis"
  - Procedure code matches a CPT code in Table 2, ICD-9-CM code in Table 3, or ICD-10-PCS code in Table 4. (In other words, patient is undergoing kidney transplant or dialysis access procedure)
- Set the AKI_Dialysis variable to 1 if M_Kidney_Dialysis = 1
- Otherwise set the AKI_Dialysis variable to 0. (This includes cases where M_Kidney_Dialysis = 0 and cases where M_Kidney_Dialysis is missing.)

eTable 2. CPT Codes for Kidney Transplant and Dialysis Access Procedures

36818	Upper arm cephalic transposition
36819	Upper arm basilic vein transposition
36820	Forearm any vein transposition
36821	AV access with direct vein to artery anastomosis
36825	AV access with other than direct ateriovenous anastomosis; autogenous graft
36830	AV access with other than direct arteriovenous anastomosis; non-autogenous graft
36831	Thrombectomy of AVF, open, no revision
36832	Revision of AVF, open, no thrombectomy
36833	Revision of AVF and thrombectomy, open
50360	Renal allotransplantation; implementation of graft, excluding donor and recipient nephrectomy
50365	Renal allotransplantation, implantation of graft; with recipient nephrectomy

eTable 3. ICD-9-CM Codes for Kidney Transplant and Dialysis Access Procedures

39.27	Arteriovenostomy for renal dialysis
39.42	Revision of arteriovenous shunt for renal dialysis
39.43	Removal of arteriovenous shunt for renal dialysis
55.6	Other kidney transplantation
39.42	Revision of arteriovenous shunt for renal dialysis

eTable 4. ICD-10-PCS Codes for Kidney Transplant and Dialysis Access Procedures

0TY00Z0	Transplantation of right kidney, allogeneic, open approach

© 2021 Xue B et al. *JAMA Network Open.*

0TY00Z1	Transplantation of right kidney, syngeneic, open approach
0TY10Z0	Transplantation of left kidney, allogeneic, open approach
0TY10Z1	Transplantation of left kidney, syngeneic, open approach
031209D	Bypass Innominate Artery to Upper Arm Vein with Autologous Venous Tissue, Open Approach
031209F	Bypass Innominate Artery to Lower Arm Vein with Autologous Venous Tissue, Open Approach
03120AD	Bypass Innominate Artery to Upper Arm Vein with Autologous Arterial Tissue, Open Approach
03120AF	Bypass Innominate Artery to Lower Arm Vein with Autologous Arterial Tissue, Open Approach
03120JD	Bypass Innominate Artery to Upper Arm Vein with Synthetic Substitute, Open Approach
03120JF	Bypass Innominate Artery to Lower Arm Vein with Synthetic Substitute, Open Approach
03120KD	Bypass Innominate Artery to Upper Arm Vein with Nonautologous Tissue Substitute, Open Approach
03120KF	Bypass Innominate Artery to Lower Arm Vein with Nonautologous Tissue Substitute, Open Approach
03120ZD	Bypass Innominate Artery to Upper Arm Vein, Open Approach
03120ZF	Bypass Innominate Artery to Lower Arm Vein, Open Approach
031309D	Bypass Right Subclavian Artery to Upper Arm Vein with Autologous Venous Tissue, Open Approach
031309F	Bypass Right Subclavian Artery to Lower Arm Vein with Autologous Venous Tissue, Open Approach
03130AD	Bypass Right Subclavian Artery to Upper Arm Vein with Autologous Arterial Tissue, Open Approach
03130AF	Bypass Right Subclavian Artery to Lower Arm Vein with Autologous Arterial Tissue, Open Approach
03130JD	Bypass Right Subclavian Artery to Upper Arm Vein with Synthetic Substitute, Open Approach
03130JF	Bypass Right Subclavian Artery to Lower Arm Vein with Synthetic Substitute, Open Approach
03130KD	Bypass Right Subclavian Artery to Upper Arm Vein with Nonautologous Tissue Substitute, Open Approach
03130KF	Bypass Right Subclavian Artery to Lower Arm Vein with Nonautologous Tissue Substitute, Open Approach
03130ZD	Bypass Right Subclavian Artery to Upper Arm Vein, Open Approach

© 2021 Xue B et al. *JAMA Network Open.*

03130ZF	Bypass Right Subclavian Artery to Lower Arm Vein, Open Approach
031409D	Bypass Left Subclavian Artery to Upper Arm Vein with Autologous Venous Tissue, Open Approach
031409F	Bypass Left Subclavian Artery to Lower Arm Vein with Autologous Venous Tissue, Open Approach
03140AD	Bypass Left Subclavian Artery to Upper Arm Vein with Autologous Arterial Tissue, Open Approach
03140AF	Bypass Left Subclavian Artery to Lower Arm Vein with Autologous Arterial Tissue, Open Approach
03140JD	Bypass Left Subclavian Artery to Upper Arm Vein with Synthetic Substitute, Open Approach
03140JF	Bypass Left Subclavian Artery to Lower Arm Vein with Synthetic Substitute, Open Approach
03140KD	Bypass Left Subclavian Artery to Upper Arm Vein with Nonautologous Tissue Substitute, Open Approach
03140KF	Bypass Left Subclavian Artery to Lower Arm Vein with Nonautologous Tissue Substitute, Open Approach
03140ZD	Bypass Left Subclavian Artery to Upper Arm Vein, Open Approach
03140ZF	Bypass Left Subclavian Artery to Lower Arm Vein, Open Approach
031509D	Bypass Right Axillary Artery to Upper Arm Vein with Autologous Venous Tissue, Open Approach
031509F	Bypass Right Axillary Artery to Lower Arm Vein with Autologous Venous Tissue, Open Approach
03150AD	Bypass Right Axillary Artery to Upper Arm Vein with Autologous Arterial Tissue, Open Approach
03150AF	Bypass Right Axillary Artery to Lower Arm Vein with Autologous Arterial Tissue, Open Approach
03150JD	Bypass Right Axillary Artery to Upper Arm Vein with Synthetic Substitute, Open Approach
03150JF	Bypass Right Axillary Artery to Lower Arm Vein with Synthetic Substitute, Open Approach
03150KD	Bypass Right Axillary Artery to Upper Arm Vein with Nonautologous Tissue Substitute, Open Approach
03150KF	Bypass Right Axillary Artery to Lower Arm Vein with Nonautologous Tissue Substitute, Open Approach
03150ZD	Bypass Right Axillary Artery to Upper Arm Vein, Open Approach

© 2021 Xue B et al. *JAMA Network Open*.

03150ZF	Bypass Right Axillary Artery to Lower Arm Vein, Open Approach
031609D	Bypass Left Axillary Artery to Upper Arm Vein with Autologous Venous Tissue, Open Approach
031609F	Bypass Left Axillary Artery to Lower Arm Vein with Autologous Venous Tissue, Open Approach
03160AD	Bypass Left Axillary Artery to Upper Arm Vein with Autologous Arterial Tissue, Open Approach
03160AF	Bypass Left Axillary Artery to Lower Arm Vein with Autologous Arterial Tissue, Open Approach
03160JD	Bypass Left Axillary Artery to Upper Arm Vein with Synthetic Substitute, Open Approach
03160JF	Bypass Left Axillary Artery to Lower Arm Vein with Synthetic Substitute, Open Approach
03160KD	Bypass Left Axillary Artery to Upper Arm Vein with Nonautologous Tissue Substitute, Open Approach
03160KF	Bypass Left Axillary Artery to Lower Arm Vein with Nonautologous Tissue Substitute, Open Approach
03160ZD	Bypass Left Axillary Artery to Upper Arm Vein, Open Approach
03160ZF	Bypass Left Axillary Artery to Lower Arm Vein, Open Approach
031709D	Bypass Right Brachial Artery to Upper Arm Vein with Autologous Venous Tissue, Open Approach
031709F	Bypass Right Brachial Artery to Lower Arm Vein with Autologous Venous Tissue, Open Approach
03170AD	Bypass Right Brachial Artery to Upper Arm Vein with Autologous Arterial Tissue, Open Approach
03170AF	Bypass Right Brachial Artery to Lower Arm Vein with Autologous Arterial Tissue, Open Approach
03170JD	Bypass Right Brachial Artery to Upper Arm Vein with Synthetic Substitute, Open Approach
03170JF	Bypass Right Brachial Artery to Lower Arm Vein with Synthetic Substitute, Open Approach
03170KD	Bypass Right Brachial Artery to Upper Arm Vein with Nonautologous Tissue Substitute, Open Approach
03170KF	Bypass Right Brachial Artery to Lower Arm Vein with Nonautologous Tissue Substitute, Open Approach
03170ZD	Bypass Right Brachial Artery to Upper Arm Vein, Open Approach
03170ZF	Bypass Right Brachial Artery to Lower Arm Vein, Open Approach

© 2021 Xue B et al. *JAMA Network Open.*

031809D	Bypass Left Brachial Artery to Upper Arm Vein with Autologous Venous Tissue, Open Approach
031809F	Bypass Left Brachial Artery to Lower Arm Vein with Autologous Venous Tissue, Open Approach
03180AD	Bypass Left Brachial Artery to Upper Arm Vein with Autologous Arterial Tissue, Open Approach
03180AF	Bypass Left Brachial Artery to Lower Arm Vein with Autologous Arterial Tissue, Open Approach
03180JD	Bypass Left Brachial Artery to Upper Arm Vein with Synthetic Substitute, Open Approach
03180JF	Bypass Left Brachial Artery to Lower Arm Vein with Synthetic Substitute, Open Approach
03180KD	Bypass Left Brachial Artery to Upper Arm Vein with Nonautologous Tissue Substitute, Open Approach
03180KF	Bypass Left Brachial Artery to Lower Arm Vein with Nonautologous Tissue Substitute, Open Approach
03180ZD	Bypass Left Brachial Artery to Upper Arm Vein, Open Approach
03180ZF	Bypass Left Brachial Artery to Lower Arm Vein, Open Approach
031909F	Bypass Right Ulnar Artery to Lower Arm Vein with Autologous Venous Tissue, Open Approach
03190AF	Bypass Right Ulnar Artery to Lower Arm Vein with Autologous Arterial Tissue, Open Approach
03190JF	Bypass Right Ulnar Artery to Lower Arm Vein with Synthetic Substitute, Open Approach
03190KF	Bypass Right Ulnar Artery to Lower Arm Vein with Nonautologous Tissue Substitute, Open Approach
03190ZF	Bypass Right Ulnar Artery to Lower Arm Vein, Open Approach
031A09F	Bypass Left Ulnar Artery to Lower Arm Vein with Autologous Venous Tissue, Open Approach
031A0AF	Bypass Left Ulnar Artery to Lower Arm Vein with Autologous Arterial Tissue, Open Approach
031A0JF	Bypass Left Ulnar Artery to Lower Arm Vein with Synthetic Substitute, Open Approach
031A0KF	Bypass Left Ulnar Artery to Lower Arm Vein with Nonautologous Tissue Substitute, Open Approach
031A0ZF	Bypass Left Ulnar Artery to Lower Arm Vein, Open Approach
031B09F	Bypass Right Radial Artery to Lower Arm Vein with Autologous Venous Tissue, Open Approach

© 2021 Xue B et al. *JAMA Network Open.*

031B0AF	Bypass Right Radial Artery to Lower Arm Vein with Autologous Arterial Tissue, Open Approach
031B0JF	Bypass Right Radial Artery to Lower Arm Vein with Synthetic Substitute, Open Approach
031B0KF	Bypass Right Radial Artery to Lower Arm Vein with Nonautologous Tissue Substitute, Open Approach
031B0ZF	Bypass Right Radial Artery to Lower Arm Vein, Open Approach
031C09F	Bypass Left Radial Artery to Lower Arm Vein with Autologous Venous Tissue, Open Approach
031C0AF	Bypass Left Radial Artery to Lower Arm Vein with Autologous Arterial Tissue, Open Approach
031C0JF	Bypass Left Radial Artery to Lower Arm Vein with Synthetic Substitute, Open Approach
031C0KF	Bypass Left Radial Artery to Lower Arm Vein with Nonautologous Tissue Substitute, Open Approach
031C0ZF	Bypass Left Radial Artery to Lower Arm Vein, Open Approach
03PY07Z	Removal of Autologous Tissue Substitute from Upper Artery, Open Approach
03PY0JZ	Removal of Synthetic Substitute from Upper Artery, Open Approach
03PY0KZ	Removal of Nonautologous Tissue Substitute from Upper Artery, Open Approach
03PY37Z	Removal of Autologous Tissue Substitute from Upper Artery, Percutaneous Approach
03PY3JZ	Removal of Synthetic Substitute from Upper Artery, Percutaneous Approach
03PY3KZ	Removal of Nonautologous Tissue Substitute from Upper Artery, Percutaneous Approach
03PY47Z	Removal of Autologous Tissue Substitute from Upper Artery, Percutaneous Endoscopic Approach
03PY4JZ	Removal of Synthetic Substitute from Upper Artery, Percutaneous Endoscopic Approach
03PY4KZ	Removal of Nonautologous Tissue Substitute from Upper Artery, Percutaneous Endoscopic Approach

SATISFY-SOS Pilot Study – Algorithm for Automated Medical Record Review for all other Postoperative Complications

TASK 1: Identify the target date range

A. Variables to be used in this procedure
    a. **OR_Date** – This is the date that triggered our team to send a survey to the patient. It is defined as the first time the patient received a billable anesthesia service at a BJC facility starting two weeks prior to the date of study consent. It is often, but not always, the same as the procedure of interest. There are no missing values.

© 2021 Xue B et al. *JAMA Network Open.*

b. **PAP_Type** – This is a categorical variable indicating where the patient underwent preoperative assessment by the anesthesiology department. Possible values include
   i. "CPAP Clinic" – assessed at Center for Preoperative Assessment and Planning, an outpatient clinic
   ii. "CPAP-incomplete" – assessed at CPAP, but data form is <75% complete
   iii. "DPAP (holding area)" – assessed on day of surgery in preop holding area
   iv. "DPAP (on ward)" – assessed on day of surgery on hospital ward
   v. "IPAP" – assessed as an inpatient, prior to day of surgery
   vi. "IPAP-incomplete" – assessed as inpatient, but data form is <75% complete
   vii. "TPAP-AP" – assessed by telephone
   viii. "TPAP-RN" – assessed by telephone by a nurse

c. **PAP_Date** – This is the date that the patient underwent their CPAP, DPAP, IPAP, or TPAP. In most cases, this is also the date of study consent.

d. **CPAP_DOSPlanned** – This is the date of anticipated surgery, as documented in the preoperative assessment note. The value is missing for about 50% of patients in our study. If the patient has a surgery on this date, it is likely the procedure of interest.

B. Identify the date of the procedure of interest

a. If PAP_Type = "IPAP" or "IPAP-incomplete" or "DPAP (holding area)" or "DPAP (on ward)" or "TPAP-AP" or "TPAP-RN" *<u>OR</u>* if PAP_Type = [missing]

   i. Then **OR_Date** gives the date of the procedure of interest.
   Patients who did not go the CPAP clinic must have been consented during the hospitalization for the procedure of interest. It is safe to assume that these patients have not had additional billable anesthesia services in the past two weeks that were not associated with the current hospitalization. Therefore OR_Date accurately gives the date of the procedure of interest.

b. If PAP_Type = "CPAP Clinic" or "CPAP-incomplete" *<u>AND</u>* OR_Date = CPAP_DOSPlanned

   i. Then **OR_Date** gives the date of the procedure of interest.
   These criteria select patients who were seen in CPAP, had the procedure of interest on the originally scheduled date, and did not undergo any minor procedure between the CPAP visit and the procedure of interest.

c. **If PAP_Type = "CPAP Clinic" or "CPAP-incomplete" *<u>AND</u>* OR_Date ≠ CPAP_DOSPlanned** (including cases where CPAP_DOSPlanned = [missing])
These criteria select patients with any of the following conditions: (1) Patient had a minor procedure, and then had their planned procedure of interest on the originally scheduled day. (2) Patient had a minor procedure, and then had their planned procedure of interest on a day other than the originally scheduled day. (3) Patient had no minor procedure, but had their planned procedure of interest

© 2021 Xue B et al. *JAMA Network Open.*

on a day other than the originally scheduled day. (4) Patient had a minor procedure, and did not have their planned procedure of interest.

i. Then search for "**Anesthesia Record**" document with a date matching "**CPAP_DOSPlanned**". If there is a match, then "CPAP_DOSPlanned" gives the date of the procedure of interest. If there is no match, continue to the next step. If "CPAP_DOSPlanned" is missing, skip this step. *This covers situation (1) above.*

ii. If neither of the above searches yields a result, then use "**OR_Date**" as the date of the procedure of interest. *This covers situations (2), (3), and (4) above. For situation (2), we have identified the incorrect procedure but do not have sufficient information to find the procedure of interest. For situation (3), we have identified the procedure of interest. For situation (4), realize that the patient never had the intended procedure of interest, so chart review will focus on the minor procedure.*

C. Identify the **visit number** (starts with 701… in most cases) associated with the date of the procedure of interest. This is included in the "Anesthesia Record" document, which all patients in the study will have. It is also in the operative summary document, but some patients in our study undergo procedures (i.e. electrophysiology) that do not generate that document.

D. **Restrict search** for complications to data corresponding **to this visit number**. Examine complications that occurred during hospitalization for the procedure of interest.

TASK 2: Identify complications occurring within the target date range

For all subsequent steps, restrict the search to data corresponding to the visit number identified in Task 1. In general, searches of ICD-9 diagnosis codes should exclude the admitting diagnosis, as the admitting diagnosis should be the indication for surgery and would be unlikely to represent a postoperative complication. Note that the admitting diagnosis is typically repeated in the list of final diagnoses. It should still be excluded from searches.

Variables to be used in this procedure:

- ***AFIB*** *– This is a dichotomous variable (check box) from the preoperative assessment form, indicating whether the patient reports a history of atrial fibrillation or atrial flutter. Possible values are 1 (yes) and 0 (no). If there are missing values, assume a value of 0.*
- ***AFIB_Rhythm*** *– This is a categorical variable (select from menu) from the preoperative assessment form, indicating the patient's current heart rhythm. Possible values include "atrial fibrillation," "atrial flutter," "non-fibrillation/flutter," and "indeterminate." This variable is only defined if AFIB = 1.*
- ***CKD*** *– This is a dichotomous variable (check box) from the preoperative assessment form, indicating whether the patient reports a history of chronic kidney disease. Possible values are 1 (yes) and 0 (no). If there are missing values, assume a value of 0.*
- ***CKD_DialysisHistory*** *– This is a categorical variable (select from menu) from the preoperative assessment form, indicating if the patient is currently receiving dialysis or*

© 2021 Xue B et al. *JAMA Network Open.*

has done so previously. Possible values include "never," "ongoing peritoneal dialysis," "ongoing hemodialysis," and "past dialysis." This variable is only defined if CKD = 1.

A. **Blood clot in your leg**
Chronic deep venous thrombosis and venous thrombosis outside of the leg are excluded. Chart review is positive for this complication if this criterion is met:
   a. ANY of the following ICD-9 codes are listed as a final diagnosis and not listed as the admitting diagnosis
      i. 453.4X (Acute venous embolism and thrombosis of deep vessels of lower extremity)

B. **Blood clot in your lung**
Chart review is positive for this complication if this criterion is met:
   a. ANY of the following ICD-9 codes are listed as a final diagnosis and not listed as the admitting diagnosis
      i. 415.1X (Pulmonary embolism and infarction)

C. **An infection in your lungs (pneumonia)**
Chart review is positive for this complication if this criterion is met:
   a. ANY of the following ICD-9 codes are listed as a final diagnosis and not listed as the admitting diagnosis
      i. 011.6X (Tuberculous pneumonia)
      ii. 073.0 (Ornithosis with pneumonia)
      iii. 112.4 (Candidiasis of lung)
      iv. 136.3 (Pneumocystosis)
      v. 480.X (Viral pneumonia)
      vi. 481 (Pneumococcal pneumonia)
      vii. 482.X (Other bacterial pneumonia)
      viii. 483.X (Pneumonia due to other specified organism)
      ix. 484.X (Pneumonia in infectious diseases classified elsewhere)
      x. 485 (Bronchopneumonia, organism unspecified)
      xi. 486 (Pneumonia, organism unspecified)
      xii. 487.0 (Influenza with pneumonia)
      xiii. 510.X (Empyema)
      xiv. 997.31 (Ventilator associated pneumonia)
      xv. 997.32 (Postprocedural aspiration pneumonia)

D. Kidney failure and you needed dialysis
*Chart review is positive for this complication if **BOTH** of the following criteria are met:*
   a. ANY of the following ICD-9 codes are listed as a procedure
      i. 39.95 (Hemodialysis)
      ii. 54.98 (Peritoneal dialysis)
   b. The patient is not a home dialysis patient. Must meet AT LEAST ONE of the following criteria
      i. CKD = 0 or [missing]
      The patient did not have known kidney disease preoperatively.

© 2021 Xue B et al. *JAMA Network Open.*

ii. CKD = 1 *AND* CKD_DialysisHistory = “Never” or “Past dialysis” or [missing]
The patient had known kidney disease preoperatively, but was not receiving ongoing hemodialysis or peritoneal dialysis.

E. **Delirium**

Chart review is positive for this complication if this criterion is met:

a. ANY of the following ICD-9 codes are listed as a final diagnosis and not listed as the admitting diagnosis
   i. 290.11 (Presenile dementia with delirium)
   ii. 290.3 (Senile dementia, with delirium)
   iii. 290.41 (Vascular dementia, with delirium)
   iv. 291.0 (Alcohol withdrawal delirium)
   v. 292.81 (Drug-induced delirium)
   vi. 293.0 (Delirium due to conditions classified elsewhere)
   vii. 293.1 (Subacute delirium)

© 2021 Xue B et al. *JAMA Network Open.*

eAppendix 2. List of Variables and Data Type

eTable 5. The list of preoperative variables, missing rates, total records, and data type

**Variable**	**Availability rate**	**Total records**	**Count of missing data**	**Data Type**
Hypertension	100.00%	111929	0	binary
Coronary Arterial Disease	100.00%	111929	0	binary
History of Myocardial Infraction	100.00%	111929	0	binary
Congestive Heart Failure	100.00%	111929	0	binary
Permanent Pacemaker	100.00%	111929	0	binary
History of Stroke	100.00%	111929	0	binary
Peripheral Artery Disease	100.00%	111929	0	binary
Deep Venous Thrombosis	100.00%	111929	0	binary
Pulmonary Embolism	100.00%	111929	0	binary
Diabetes Mellitus	100.00%	111929	0	binary
Chronic Kidney Disease	100.00%	111929	0	binary
Pulmonary Hypertension	100.00%	111929	0	binary
Chronic Obstructive Pulmonary Disease	100.00%	111929	0	binary
Asthma	100.00%	111929	0	binary
Obstructive Sleep Apnea	100.00%	111929	0	binary
Cirrhosis	100.00%	111929	0	binary
Cancer History	100.00%	111929	0	binary
Gastroesophageal Reflux Disease	100.00%	111929	0	binary
Anemia	100.00%	111929	0	binary
Coombs Positive	100.00%	111929	0	binary
Dementia	100.00%	111929	0	binary
Peptic Ulcer	100.00%	111929	0	binary
LVEF (Left ventricular ejection fraction)	99.99%	111918	11	discrete [31, 38]
Outpatient Insulin Use	99.98%	111910	19	binary
ASA	99.98%	111902	27	discrete [1,6]
Valvular Disease	99.90%	111819	110	discrete [60, 65]
Left Ventricular Diastolic Function	99.71%	111604	325	discrete [20. 23]
Dialysis History	99.64%	111529	400	binary
Sex	99.04%	110858	1071	categorical
Age	99.03%	110846	1083	continuous
Preop Heart Rate	98.94%	110743	1186	continuous
Preop Spo2	98.89%	110684	1245	continuous
Weight	98.82%	110611	1318	continuous
CCI	98.48%	110223	1706	discrete [0,15]
Race	95.33%	106705	5224	categorical
Height	93.98%	105187	6742	continuous
BMI	93.91%	105113	6816	continuous
Ideal Body Weight	93.09%	104191	7738	continuous
Smoking Habits	91.29%	102185	9744	binary

© 2021 Xue B et al. *JAMA Network Open.*

Glucose	76.54%	85676	26253	continuous
Hematocrit	75.83%	84878	27051	continuous
Sodium	75.01%	83959	27970	continuous
Potassium	75.00%	83951	27978	continuous
Urea Nitrogen	74.88%	83818	28111	continuous
Creatinine	73.91%	82728	29201	continuous
White Blood Cells	73.81%	82612	29317	continuous
Preop Systolic	42.58%	47655	64274	continuous
Preop Diastolic	42.57%	47649	64280	continuous
Functional Capacity	42.24%	47284	64645	discrete [6, 9]
Partial Thromboplastin Time	39.82%	44574	67355	continuous
Planned Surgery Type	39.07%	43736	68193	categorical
Albumin	34.18%	38256	73673	continuous
Alkaline Phosphatase	33.88%	37917	74012	continuous
Alanine Aminotransferase Level	33.87%	37908	74021	continuous
History of Delirium	11.54%	12919	99010	binary
Atrial Fibrillation	2.09%	2340	109589	discrete (4,7]
Platelets	1.76%	1967	109962	continuous

eTable6: The list of intraoperative variables, missing rates and total records

Variable Name	Number of Patients	Availability Rate
SpO2	109819	98.11%
Heart Rate	109816	98.11%
Respiration rate	109796	98.09%
Pulse	109735	98.04%
O2 inspiratory concentration	109157	97.52%
ETCO2 (mmHg)	109009	97.39%
Systolic Blood Pressure non-invasive	106672	95.30%
Diastolic blood pressure non-invasive	106665	95.30%
Mean blood pressure - non-invasive	106599	95.24%
Peak inspiratory pressure	102907	91.94%
Tidal Volume	98227	87.76%
BJ Temp (Centigrade)	98186	87.72%
Respiratory Minute Volume	97602	87.20%
TOTALMAC(minimum alveolar concentration)	97390	87.01%
TOTALMACAGEADJ (TOTALMAC normalized to patient’s age)	97390	87.01%
PEEP	96169	85.92%
Urine output	73711	65.86%

© 2021 Xue B et al. *JAMA Network Open.*

Sevoflurane expiratory concentration	68853	61.51%
Sevoflurane inspiratory concentration	68763	61.43%
Phenylephrine	66058	59.02%
Estimated blood loss	61004	54.50%
Desflurane expiratory concentration	52779	47.15%
Desflurane inspiratory concentration	52678	47.07%
Plateau pressure	33805	30.21%
N2O expiratory concentration	31762	28.38%
Phenylephrine fluid	30950	27.68%
Mean Blood Pressure - Invasive	26592	23.83%
N2O inspiratory concentration	26204	23.50%
BJ N Systolic Blood Pressure invasive	25950	23.41%
Diastolic blood pressure invasive	25846	23.32%
Norepinephrine_fluid	9026	8.15%
Central Venous Pressure	8951	8.09%
Norepinephrine	7529	6.81%
Bispectral Index	7395	6.71%
BIS EMG (Detected muscle activity from a BIS probe)	7192	6.74%
HCT (Hematocrit)	7017	6.67%
POTASSIUM	6982	6.64%
Epinephrine	6429	6.17%
BIS SEF (Spectral edge frequency measured from a BIS probe)	6235	6.10%
Isoflurane expiratory concentration	6207	7.24%
Isoflurane inspiratory concentration	6136	7.23%
BIS SR (Burst suppression measured from a BIS probe)	5536	6.59%
SECSURPPRESSED (Transformation of BIS SR)	5564	6.63%
PCO2 Arterial POC	5390	6.43%
PH Arterial POC	5390	6.52%
PO2 Arterial POC	5390	6.52%
Glucose Arterial POC	5376	11.28%

Bicarb Arterial POC	5214	10.94%
Base Excess Arterial POC	5139	10.87%
Dobutamine fluid	2866	6.43%
Vasopressin fluid	2779	6.35%
Epinephrine fluid	2226	5.82%
GLUCOSE Venous POC	1743	4.60%
PLATELET	1499	3.95%
BJ T Core	1151	8.91%
INR (International Normalized Ratio, a measure of blood coagulation function)	522	22.31%
BIS TP (Total power measured from a BIS probe)	1	0.05%

© 2021 Xue B et al. *JAMA Network Open.*

eAppendix 3. Exploration on Data Imputation

eMethods 2

In this section, we have conducted comparison between 7 most common data imputation techniques: mode, mean, median, dummy indication, Multiple Imputation by Chained Equations[2] (with 10 iterations), MissForest[3] (with 10 iterations), and kNN imputation. Due to the selection of hyper-parameters in kNN, we further implemented kNN with number of nearest neighbors =3 and uniform weights, and number of nearest neighbors =5 and distance-based weights. In total, 8 imputation methods were compared.

The experiment was designed using pneumonia dataset in 3 steps. First, each imputation method was performed on a copy of the original pneumonia dataset, and the imputed values for the nominal (categorical) variables were rounded to the closest values in the original distribution. By doing so, the range of each variable was reserved. With 8 imputation methods implemented, we ended up with 8 imputed datasets. Second, each imputed dataset was processed in the same way as described in the manuscript. The categorical variables were split into binary variables by one-hot-encoding, and continuous variables were normalized by z-scoring. Last, each processed dataset was evaluated by gradient boosting tree (GBT), random forest (RF) and logistic regression (LR) with 5 random shuffles of cross validation. The configuration of GBT, RF and LR was the same as in the manuscript.

The performance of each imputation method was tabulated in eTable 1. As clearly shown in the table, regardless of imputation methods that we used, GBT had the best performance in terms of both AUROC and AUPRC. Moreover, regardless of any machine learning models used, we observed that the imputed dataset using dummy indicator was most predictive. This might be explained by the fact that some measurements, especially lab tests, are missing by "intention": when clinicians decide not to perform a lab test on a patient, it reflects the clinicians' opinion that the lab result is expected to be normal. As a result, the indication of missingness in dummy indicator method preserves this "intention" of clinicians, hence the imputed dataset becomes more informative.

eTable7: The comparison of imputation methods

Imputation Method	AUROC	AUPRC
**GBT**		
Dummy Indicator	0.905 (0.903,0.907)	0.208 (0.203,0.213)
Mean	0.901 (0.898,0.904)	0.206 (0.199,0.213)
Median	0.901 (0.898,0.904)	0.204 (0.199,0.210)
Mode	0.902 (0.900,0.904)	0.203 (0.198,0.208)
kNN-3	0.884 (0.882,0.887)	0.182 (0.176,0.188)
kNN-5	0.884 (0.882,0.887)	0.182 (0.176,0.188)
MICE	0.893 (0.890,0.896)	0.193 (0.187,0.200)
MissForest	0.890 (0.888,0.893)	0.184 (0.178,0.189)
**LR**		
Dummy Indicator	0.896 (0.892,0.899)	0.187 (0.181,0.193)
Mean	0.88 (0.877,0.883)	0.177 (0.171,0.182)
Median	0.884 (0.881,0.887)	0.178 (0.173,0.184)
Mode	0.89 (0.887,0.892)	0.181 (0.175,0.186)
kNN-3	0.872 (0.869,0.874)	0.161 (0.156,0.166)

© 2021 Xue B et al. *JAMA Network Open.*

kNN-5	0.871 (0.868,0.874)	0.161 (0.156,0.165)
MICE	0.878 (0.875,0.880)	0.16 (0.157,0.163)
MissForest	0.879 (0.877,0.882)	0.161 (0.157,0.165)
**RF**		
Dummy Indicator	0.892 (0.889,0.894)	0.17 (0.165,0.175)
Mean	0.887 (0.884,0.890)	0.168 (0.163,0.173)
Median	0.886 (0.884,0.889)	0.169 (0.163,0.174)
Mode	0.887 (0.884,0.890)	0.167 (0.162,0.172)
kNN-3	0.867 (0.864,0.869)	0.145 (0.139,0.152)
kNN-5	0.867 (0.864,0.869)	0.145 (0.139,0.152)
MICE	0.871 (0.868,0.874)	0.149 (0.142,0.155)
MissForest	0.836 (0.764,0.908)	0.135 (0.123,0.148)

© 2021 Xue B et al. *JAMA Network Open.*

eAppendix 4. Model Development

eMethods 3

For support vector machine (SVM), the regularizer was set to l2 norm to avoid overfitting and loss function was set to squared hinge loss. Due to the large number of records in the dataset, a linear SVM was used. In logistic regression (LR), Newton-CG solver was used for its optimal performance in large datasets. The hyper-parameters of random forest (RF) and deep neural network (DNN) were chosen by grid search. In RF, we varied the number of base learners from 40 to 300, maximum depth from 20 to 200, and minimum samples for splits from 1 to 7. The optimal hyper parameters for RF were 300 base learners, 200 maximum depth, and minimum 4 samples for splits. For DNN models, we explored both 3-layer, 4-layer and 5-layer architecture by varying number of nodes in each layer. When exploring the optimal DNN architecture, we varied the number of nodes in the first layer as 16, 32, 64, 128. The number of nodes in the second layer was chosen as half of the first layer accordingly, the number of nodes in third layer was chosen as half of the second layer, and so on. The last layer of DNN model was always unchanged and had 2 nodes, as it directly connected to the softmax layer to generate probabilistic output. The optimal configuration of DNN had 4 layers with 128, 64, 32, and 2 nodes in each layer). When training the DNN model, we further explored the choice of learning rate as 0.0001, 0.001, 0.01 and different batch size options as 32, 64, 128, 256, 512, 1024, 2048. The optimal settings are choosing learning rate as 0.001 and batch size as 2048. Gradient boosting tree (GBT) was created by setting tree-based learners and logistic loss function. Note that different versions of GBT may affect model performance, due to parameter setting. Version list and Python codes are uploaded to Github: xuebing1234/handoff_framework

eAppendix 5. Details of Performance Metrics of Each Model

Note that sensitivity, specificity, prevision, F-score and accuracy vary depending on the threshold of ML models (as shown in ROC curves), we fixed specificity at 95% for easier comparison between different models. As shown in eTable 1 to eTable 5, in most cases the machine learning model with highest AUROC would have the highest AUPRC too, hence model selection based on either AUROC or AUPRC would yield similar results. Such observation is consistent with theory, that if a model dominates in ROC curve, then it also dominates in PRC curve[1]. See reference for detailed proof. When the model is determined, the thresholds can be carefully adjusted based on the clinicians' judgement on the relative weight between sensitivity, specificity, etc.

eTable8: AUROCs of best machine learning models for pneumonia, acute kidney injury (AKI), deep vein thrombosis (DVT), pulmonary embolism (PE) and delirium.

**Pneumonia**		**AKI**		**DVT**		**PE**		**Delirium**	
Intraoperative Only									
Gradient Boosting Tree	0.861 (0.858,0.864)	Gradient Boosting Tree	0.799 (0.797,0.802)	Gradient Boosting Tree	0.809 (0.804,0.813)	Deep Neural Network	0.734 (0.728,0.740)	Gradient Boosting Tree	0.738 (0.735,0.741)
Preoperative Only									
Gradient Boosting Tree	0.886 (0.883,0.888)	Random Forest	0.816 (0.813,0.818)	Gradient Boosting Tree	0.865 (0.861,0.868)	Support Vector Machine	0.822 (0.811,0.833)	Random Forest	0.76 (0.755,0.765)
Combined									
Gradient Boosting Tree	0.905 (0.903,0.907)	Gradient Boosting Tree	0.848 (0.846,0.851)	Gradient Boosting Tree	0.881 (0.878,0.884)	Deep Neural Network	0.831 (0.824,0.839)	Gradient Boosting Tree	0.762 (0.759,0.765)

eTable9: Detailed performance of 5 machine learning models for pneumonia: GBT: Gradient Boosting Tree; LR: Logistic Regression; RF: Random Forest; DNN: Deep Neural Network; SVM: Support Vector Machine.

**Model**	**AUROC**	**AUPRC**	**Sensitivity**	**Specificity**	**Precision**	**F-score**	**Accuracy**

© 2021 Xue B et al. *JAMA Network Open.*

InOp							
**GBT**	0.861 (0.858,0.864)	0.149 (0.147,0.152)	0.412 (0.404,0.420)	0.95 (0.950,0.951)	0.154 (0.151,0.156)	0.224 (0.220,0.228)	0.938 (0.938,0.939)
LR	0.852 (0.849,0.856)	0.132 (0.128,0.136)	0.386 (0.377,0.396)	0.95 (0.950,0.950)	0.145 (0.143,0.148)	0.211 (0.207,0.215)	0.938 (0.938,0.938)
RF	0.844 (0.841,0.848)	0.113 (0.110,0.116)	0.341 (0.330,0.351)	0.95 (0.950,0.951)	0.131 (0.128,0.134)	0.189 (0.185,0.194)	0.937 (0.937,0.938)
DNN	0.84 (0.836,0.844)	0.107 (0.104,0.111)	0.328 (0.315,0.341)	0.95 (0.949,0.951)	0.126 (0.123,0.129)	0.182 (0.177,0.187)	0.936 (0.935,0.937)
SVM	0.85 (0.847,0.853)	0.126 (0.121,0.130)	0.379 (0.370,0.387)	0.95 (0.950,0.950)	0.143 (0.140,0.146)	0.208 (0.204,0.212)	0.938 (0.937,0.938)
PreOp							
**GBT**	0.886 (0.883,0.888)	0.156 (0.150,0.161)	0.473 (0.460,0.486)	0.95 (0.949,0.950)	0.171 (0.167,0.175)	0.251 (0.245,0.257)	0.939 (0.939,0.940)
LR	0.882 (0.879,0.885)	0.144 (0.140,0.148)	0.446 (0.435,0.458)	0.95 (0.949,0.950)	0.163 (0.159,0.167)	0.239 (0.233,0.244)	0.939 (0.939,0.939)
RF	0.874 (0.872,0.877)	0.13 (0.127,0.133)	0.426 (0.414,0.437)	0.951 (0.950,0.951)	0.159 (0.156,0.162)	0.232 (0.227,0.236)	0.939 (0.939,0.940)
DNN	0.865 (0.862,0.869)	0.122 (0.118,0.126)	0.376 (0.358,0.395)	0.949 (0.947,0.952)	0.141 (0.138,0.144)	0.204 (0.200,0.209)	0.937 (0.935,0.939)
SVM	0.881 (0.878,0.884)	0.144 (0.140,0.148)	0.438 (0.430,0.447)	0.95 (0.950,0.951)	0.162 (0.159,0.164)	0.236 (0.232,0.240)	0.939 (0.939,0.940)
Combined							
**GBT**	0.905 (0.903,0.907)	0.208 (0.203,0.213)	0.525 (0.514,0.535)	0.95 (0.950,0.950)	0.188 (0.184,0.191)	0.277 (0.272,0.281)	0.941 (0.940,0.941)
LR	0.896 (0.892,0.899)	0.187 (0.181,0.193)	0.506 (0.493,0.518)	0.95 (0.950,0.950)	0.182 (0.178,0.185)	0.268 (0.262,0.273)	0.94 (0.940,0.941)
RF	0.892 (0.889,0.894)	0.17 (0.165,0.175)	0.456 (0.449,0.463)	0.95 (0.949,0.951)	0.168 (0.165,0.170)	0.245 (0.241,0.249)	0.939 (0.939,0.940)
DNN	0.886 (0.883,0.889)	0.142 (0.139,0.146)	0.428 (0.416,0.439)	0.949 (0.948,0.950)	0.156 (0.153,0.159)	0.228 (0.224,0.233)	0.938 (0.937,0.939)
SVM	0.893 (0.890,0.897)	0.18 (0.176,0.185)	0.493 (0.483,0.502)	0.95 (0.950,0.950)	0.179 (0.176,0.182)	0.262 (0.258,0.267)	0.94 (0.940,0.941)

© 2021 Xue B et al. *JAMA Network Open.*

eTable10: Detailed performance of 5 machine learning models for acute kidney injury: GBT: Gradient Boosting Tree; LR: Logistic Regression; RF: Random Forest; DNN: Deep Neural Network; SVM: Support Vector Machine.

Model	AUROC	AUPRC	Sensitivity	Specificity	Precision	F-score	Accuracy
InOp							
**GBT**	0.799 (0.797,0.802)	0.236 (0.231,0.240)	0.312 (0.306,0.318)	0.95 (0.950,0.951)	0.288 (0.284,0.292)	0.299 (0.294,0.304)	0.911 (0.911,0.912)
LR	0.779 (0.777,0.781)	0.204 (0.201,0.208)	0.278 (0.273,0.284)	0.95 (0.949,0.950)	0.263 (0.259,0.268)	0.271 (0.266,0.275)	0.909 (0.908,0.909)
RF	0.796 (0.793,0.798)	0.206 (0.203,0.209)	0.28 (0.274,0.285)	0.95 (0.949,0.950)	0.265 (0.262,0.269)	0.272 (0.268,0.277)	0.909 (0.909,0.910)
DNN	0.749 (0.747,0.752)	0.194 (0.191,0.197)	0.265 (0.260,0.270)	0.95 (0.950,0.951)	0.256 (0.252,0.260)	0.26 (0.256,0.265)	0.908 (0.908,0.909)
SVM	0.778 (0.776,0.779)	0.2 (0.198,0.202)	0.269 (0.264,0.273)	0.95 (0.950,0.951)	0.259 (0.256,0.262)	0.264 (0.260,0.267)	0.909 (0.908,0.909)
PreOp							
GBT	0.813 (0.811,0.816)	0.264 (0.259,0.269)	0.361 (0.353,0.368)	0.95 (0.950,0.950)	0.317 (0.313,0.321)	0.337 (0.332,0.343)	0.914 (0.914,0.915)
LR	0.801 (0.799,0.803)	0.221 (0.218,0.225)	0.317 (0.311,0.322)	0.95 (0.950,0.950)	0.29 (0.286,0.294)	0.303 (0.298,0.307)	0.912 (0.911,0.912)
**RF**	0.816 (0.813,0.818)	0.268 (0.263,0.272)	0.355 (0.348,0.361)	0.95 (0.950,0.951)	0.314 (0.311,0.318)	0.333 (0.329,0.338)	0.914 (0.914,0.915)
DNN	0.774 (0.771,0.776)	0.187 (0.185,0.190)	0.247 (0.239,0.256)	0.95 (0.948,0.952)	0.243 (0.239,0.247)	0.245 (0.240,0.249)	0.908 (0.906,0.909)
SVM	0.8 (0.798,0.802)	0.22 (0.217,0.224)	0.313 (0.307,0.318)	0.95 (0.949,0.950)	0.286 (0.283,0.289)	0.299 (0.294,0.303)	0.911 (0.911,0.912)
Combined							
**GBT**	0.848 (0.846,0.851)	0.306 (0.301,0.310)	0.405 (0.399,0.412)	0.95 (0.950,0.950)	0.344 (0.340,0.348)	0.372 (0.367,0.377)	0.917 (0.916,0.917)
LR	0.834 (0.832,0.836)	0.253 (0.250,0.256)	0.354 (0.348,0.360)	0.95 (0.950,0.951)	0.314 (0.311,0.318)	0.333 (0.329,0.337)	0.914 (0.913,0.914)
RF	0.847 (0.845,0.849)	0.285 (0.281,0.290)	0.378 (0.375,0.382)	0.95 (0.950,0.951)	0.33 (0.328,0.333)	0.353 (0.350,0.356)	0.916 (0.915,0.916)
DNN	0.812 (0.809,0.814)	0.232 (0.229,0.236)	0.308 (0.301,0.314)	0.95 (0.948,0.951)	0.283 (0.279,0.286)	0.295 (0.290,0.299)	0.91 (0.910,0.911)

© 2021 Xue B et al. *JAMA Network Open.*

SVM	0.832 (0.829,0.834)	0.25 (0.246,0.253)	0.347 (0.340,0.353)	0.95 (0.949,0.950)	0.31 (0.306,0.313)	0.327 (0.322,0.332)	0.913 (0.913,0.914)

eTable11: Detailed performance of 5 machine learning models for deep vein thrombosis: GBT: Gradient Boosting Tree; LR: Logistic Regression; RF: Random Forest; DNN: Deep Neural Network; SVM: Support Vector Machine.

Model	AUROC	AUPRC	Sensitivity	Specificity	Precision	F-score	Accuracy
InOp							
**GBT**	0.809 (0.804,0.813)	0.054 (0.052,0.056)	0.277 (0.268,0.285)	0.95 (0.949,0.950)	0.068 (0.067,0.070)	0.11 (0.107,0.112)	0.941 (0.940,0.941)
LR	0.79 (0.785,0.794)	0.049 (0.048,0.051)	0.251 (0.240,0.262)	0.95 (0.949,0.950)	0.062 (0.060,0.065)	0.1 (0.096,0.104)	0.941 (0.940,0.941)
RF	0.78 (0.777,0.784)	0.046 (0.044,0.047)	0.232 (0.219,0.245)	0.95 (0.948,0.952)	0.058 (0.056,0.060)	0.093 (0.089,0.096)	0.94 (0.939,0.942)
DNN	0.777 (0.771,0.783)	0.042 (0.040,0.043)	0.213 (0.199,0.227)	0.951 (0.948,0.953)	0.054 (0.052,0.057)	0.086 (0.083,0.090)	0.941 (0.939,0.943)
SVM	0.784 (0.779,0.790)	0.048 (0.046,0.050)	0.242 (0.229,0.255)	0.95 (0.950,0.951)	0.061 (0.058,0.064)	0.097 (0.092,0.102)	0.941 (0.940,0.941)
PreOp							
**GBT**	0.865 (0.861,0.868)	0.081 (0.078,0.084)	0.377 (0.364,0.389)	0.95 (0.949,0.950)	0.09 (0.088,0.093)	0.146 (0.142,0.150)	0.942 (0.941,0.943)
LR	0.862 (0.858,0.865)	0.078 (0.076,0.080)	0.361 (0.350,0.371)	0.95 (0.950,0.951)	0.088 (0.085,0.091)	0.141 (0.137,0.146)	0.942 (0.942,0.943)
RF	0.846 (0.841,0.850)	0.066 (0.063,0.068)	0.325 (0.313,0.336)	0.953 (0.952,0.954)	0.084 (0.081,0.087)	0.133 (0.128,0.138)	0.945 (0.944,0.945)
DNN	0.849 (0.847,0.851)	0.062 (0.060,0.064)	0.315 (0.297,0.333)	0.951 (0.948,0.954)	0.079 (0.076,0.081)	0.125 (0.122,0.129)	0.942 (0.940,0.945)
SVM	0.861 (0.858,0.864)	0.077 (0.074,0.080)	0.361 (0.348,0.374)	0.95 (0.949,0.950)	0.087 (0.084,0.090)	0.141 (0.136,0.145)	0.942 (0.942,0.943)
Combined							
**GBT**	0.881 (0.878,0.884)	0.095 (0.091,0.098)	0.423 (0.411,0.436)	0.95 (0.950,0.951)	0.102 (0.100,0.105)	0.164 (0.160,0.169)	0.943 (0.943,0.944)
LR	0.868 (0.864,0.871)	0.086 (0.082,0.089)	0.397 (0.383,0.411)	0.95 (0.950,0.950)	0.095 (0.092,0.098)	0.154 (0.149,0.159)	0.943 (0.942,0.943)

© 2021 Xue B et al. *JAMA Network Open.*

RF	0.846 (0.842,0.850)	0.069 (0.067,0.071)	0.317 (0.309,0.325)	0.95 (0.950,0.951)	0.079 (0.076,0.081)	0.126 (0.122,0.129)	0.942 (0.941,0.943)
DNN	0.868 (0.865,0.871)	0.077 (0.074,0.079)	0.373 (0.357,0.389)	0.948 (0.946,0.950)	0.087 (0.085,0.089)	0.141 (0.137,0.145)	0.94 (0.939,0.942)
SVM	0.862 (0.857,0.866)	0.082 (0.079,0.086)	0.388 (0.374,0.403)	0.95 (0.950,0.950)	0.094 (0.091,0.097)	0.151 (0.146,0.156)	0.943 (0.942,0.943)

eTable12: Detailed performance of 5 machine learning models for pulmonary embolism: GBT: Gradient Boosting Tree; LR: Logistic Regression; RF: Random Forest; DNN: Deep Neural Network; SVM: Support Vector Machine.

Model	AUROC	AUPRC	Sensitivity	Specificity	Precision	F-score	Accuracy
InOp							
GBT	0.734 (0.727,0.741)	0.015 (0.014,0.017)	0.182 (0.168,0.195)	0.95 (0.950,0.951)	0.017 (0.016,0.019)	0.032 (0.029,0.034)	0.946 (0.946,0.947)
LR	0.717 (0.708,0.727)	0.014 (0.013,0.015)	0.188 (0.176,0.201)	0.95 (0.949,0.950)	0.018 (0.017,0.019)	0.033 (0.030,0.035)	0.946 (0.946,0.947)
RF	0.686 (0.680,0.693)	0.011 (0.010,0.011)	0.131 (0.118,0.143)	0.955 (0.954,0.956)	0.014 (0.013,0.015)	0.025 (0.023,0.028)	0.951 (0.950,0.952)
**DNN**	0.734 (0.728,0.740)	0.013 (0.012,0.014)	0.158 (0.145,0.171)	0.952 (0.949,0.956)	0.016 (0.015,0.018)	0.029 (0.027,0.032)	0.949 (0.945,0.952)
SVM	0.702 (0.691,0.713)	0.014 (0.013,0.015)	0.193 (0.177,0.208)	0.95 (0.950,0.951)	0.018 (0.017,0.020)	0.034 (0.031,0.036)	0.947 (0.946,0.947)
PreOp							
GBT	0.819 (0.812,0.826)	0.036 (0.031,0.042)	0.325 (0.307,0.343)	0.95 (0.949,0.950)	0.031 (0.029,0.032)	0.056 (0.053,0.059)	0.947 (0.946,0.947)
LR	0.82 (0.811,0.830)	0.03 (0.026,0.033)	0.324 (0.311,0.336)	0.95 (0.950,0.951)	0.031 (0.030,0.032)	0.056 (0.054,0.058)	0.947 (0.947,0.948)
RF	0.786 (0.779,0.794)	0.023 (0.022,0.024)	0.297 (0.285,0.309)	0.954 (0.954,0.955)	0.031 (0.029,0.032)	0.055 (0.053,0.058)	0.951 (0.950,0.952)
DNN	0.805 (0.799,0.811)	0.02 (0.018,0.021)	0.258 (0.239,0.277)	0.949 (0.947,0.952)	0.024 (0.022,0.026)	0.044 (0.041,0.047)	0.946 (0.943,0.948)
**SVM**	0.822 (0.811,0.833)	0.028 (0.024,0.032)	0.317 (0.295,0.339)	0.95 (0.950,0.951)	0.03 (0.028,0.032)	0.055 (0.051,0.058)	0.947 (0.947,0.947)
Combined							
GBT	0.828 (0.822,0.834)	0.028 (0.025,0.030)	0.313 (0.295,0.331)	0.95 (0.950,0.951)	0.03 (0.028,0.031)	0.054 (0.051,0.057)	0.947 (0.947,0.948)

© 2021 Xue B et al. *JAMA Network Open.*

LR	0.805 (0.796,0.813)	0.029 (0.025,0.032)	0.32 (0.302,0.337)	0.95 (0.950,0.950)	0.03 (0.028,0.032)	0.055 (0.052,0.058)	0.947 (0.947,0.947)
RF	0.768 (0.758,0.778)	0.017 (0.016,0.018)	0.223 (0.207,0.238)	0.951 (0.950,0.952)	0.022 (0.020,0.023)	0.039 (0.036,0.042)	0.947 (0.947,0.948)
**DNN**	0.831 (0.824,0.839)	0.026 (0.023,0.028)	0.284 (0.265,0.303)	0.95 (0.948,0.952)	0.027 (0.025,0.028)	0.049 (0.046,0.052)	0.947 (0.945,0.949)
SVM	0.791 (0.781,0.800)	0.026 (0.024,0.028)	0.302 (0.283,0.321)	0.95 (0.949,0.950)	0.028 (0.026,0.030)	0.052 (0.048,0.055)	0.947 (0.946,0.947)

eTable13: Detailed performance of 5 machine learning models for delirium: GBT: Gradient Boosting Tree; LR: Logistic Regression; RF: Random Forest; DNN: Deep Neural Network; SVM: Support Vector Machine.

Model	AUROC	AUPRC	Sensitivity	Specificity	Precision	F-score	Accuracy
InOp							
**GBT**	0.738 (0.735,0.741)	0.759 (0.756,0.763)	0.274 (0.265,0.283)	0.95 (0.949,0.950)	0.857 (0.853,0.862)	0.415 (0.404,0.426)	0.594 (0.590,0.599)
LR	0.736 (0.733,0.740)	0.759 (0.755,0.762)	0.272 (0.265,0.279)	0.95 (0.949,0.951)	0.857 (0.854,0.860)	0.413 (0.405,0.421)	0.593 (0.590,0.597)
RF	0.735 (0.731,0.739)	0.752 (0.749,0.756)	0.256 (0.248,0.264)	0.95 (0.949,0.951)	0.85 (0.846,0.855)	0.393 (0.383,0.403)	0.585 (0.581,0.589)
DNN	0.683 (0.678,0.689)	0.707 (0.702,0.711)	0.185 (0.154,0.216)	0.952 (0.944,0.961)	0.714 (0.602,0.825)	0.293 (0.245,0.341)	0.549 (0.536,0.561)
SVM	0.732 (0.728,0.735)	0.753 (0.749,0.757)	0.259 (0.252,0.266)	0.95 (0.949,0.951)	0.851 (0.847,0.855)	0.397 (0.388,0.406)	0.587 (0.583,0.590)
PreOp							
GBT	0.739 (0.736,0.743)	0.767 (0.764,0.771)	0.285 (0.276,0.293)	0.95 (0.949,0.950)	0.862 (0.857,0.866)	0.428 (0.418,0.438)	0.6 (0.595,0.604)
LR	0.731 (0.727,0.735)	0.755 (0.751,0.759)	0.252 (0.242,0.262)	0.949 (0.949,0.950)	0.846 (0.841,0.851)	0.388 (0.375,0.400)	0.582 (0.577,0.588)
**RF**	0.76 (0.755,0.765)	0.785 (0.780,0.790)	0.309 (0.296,0.322)	0.95 (0.949,0.951)	0.872 (0.868,0.876)	0.455 (0.441,0.470)	0.613 (0.606,0.619)
DNN	0.686 (0.681,0.691)	0.7 (0.695,0.705)	0.163 (0.151,0.175)	0.95 (0.945,0.955)	0.785 (0.776,0.795)	0.269 (0.252,0.285)	0.536 (0.532,0.541)
SVM	0.732 (0.728,0.735)	0.756 (0.753,0.760)	0.254 (0.244,0.264)	0.95 (0.949,0.951)	0.849 (0.844,0.854)	0.391 (0.379,0.403)	0.584 (0.579,0.589)
Combined							

© 2021 Xue B et al. *JAMA Network Open*.

**GBT**	0.762 (0.759,0.765)	0.786 (0.783,0.789)	0.311 (0.303,0.320)	0.95 (0.949,0.950)	0.873 (0.870,0.876)	0.459 (0.449,0.468)	0.614 (0.610,0.618)
LR	0.76 (0.756,0.763)	0.784 (0.781,0.788)	0.315 (0.304,0.327)	0.949 (0.949,0.950)	0.873 (0.869,0.877)	0.463 (0.450,0.476)	0.616 (0.610,0.622)
RF	0.751 (0.748,0.755)	0.768 (0.764,0.771)	0.274 (0.265,0.283)	0.95 (0.949,0.950)	0.858 (0.854,0.862)	0.415 (0.404,0.426)	0.594 (0.590,0.599)
DNN	0.715 (0.710,0.721)	0.731 (0.725,0.737)	0.229 (0.213,0.245)	0.947 (0.944,0.950)	0.827 (0.820,0.834)	0.357 (0.338,0.376)	0.569 (0.562,0.577)
SVM	0.756 (0.752,0.761)	0.781 (0.776,0.785)	0.307 (0.298,0.316)	0.95 (0.949,0.951)	0.871 (0.868,0.875)	0.454 (0.444,0.464)	0.612 (0.607,0.617)

© 2021 Xue B et al. *JAMA Network Open.*

eAppendix 6. More on Model Interpretation

In this section, we show 2 patients with negative predicted risks and 2 patients with positive predicted risks. For the risk overview, we created three candidate graphs: a) comparison of the patient with respect to the average of patients who had pneumonia (Fig 1.a, 2.a, 3.a, and 4.a), b) comparison of the patient with respect to the average of patients who did not have pneumonia (Fig 1.b, 2.b, 3.b and 4.b), and c) comparison of patients with respect to the average of patients who had pneumonia and the average of patients who did not have (Fig 1.c, 2.c, 3.c and 4.c). For the key intraoperative variables (blood pressure in the prediction model of pneumonia), we created detailed visualizations to look into it. First, we created a nested pie chart to show how much contribution (measured by SHAP values) does each statistical feature make to the prediction (Fig 1.d, 2.d, 3.d and 4.d). In the outer circle it shows the average of all patients; and in the inner circle it shows the patient-of-interest. Second, we created a bar plot to show how much does the value of each statistical feature differentiate from the average of all patients (Fig 1.e, 2.e, 3.e and 4.e). Each statistical feature was normalized to zero mean and unit variance, therefore the magnitude reflects the relative difference from average.

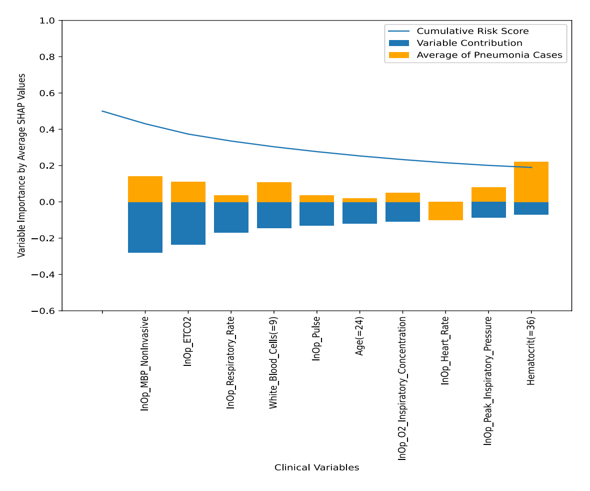

a

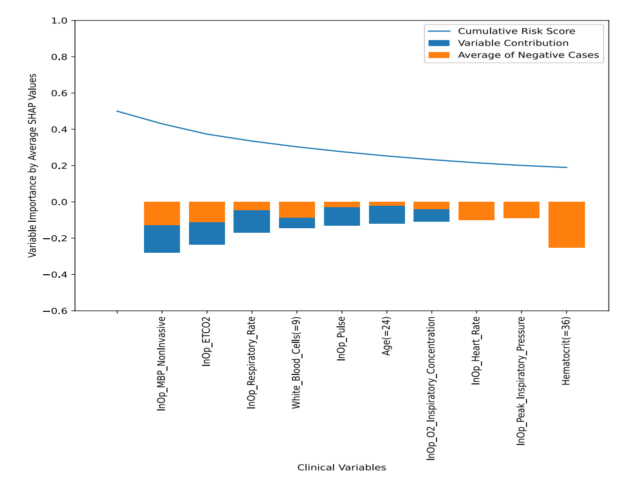

b

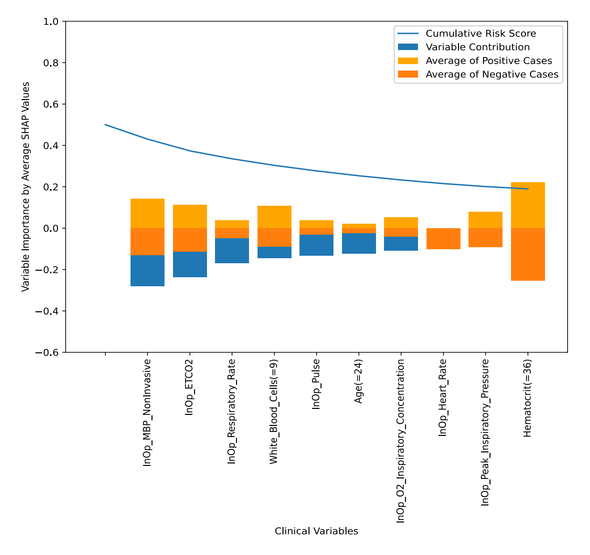

c

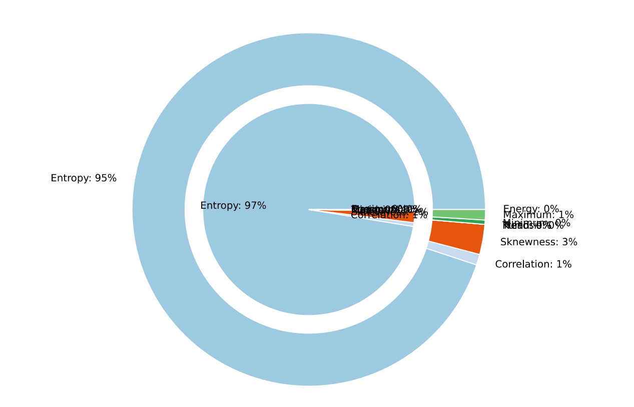

d

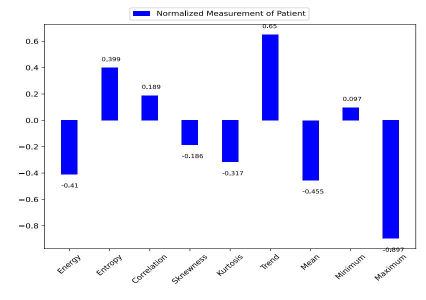

e

eFigure 1: Patient 1 - negative prediction of pneumonia

© 2021 Xue B et al. *JAMA Network Open.*

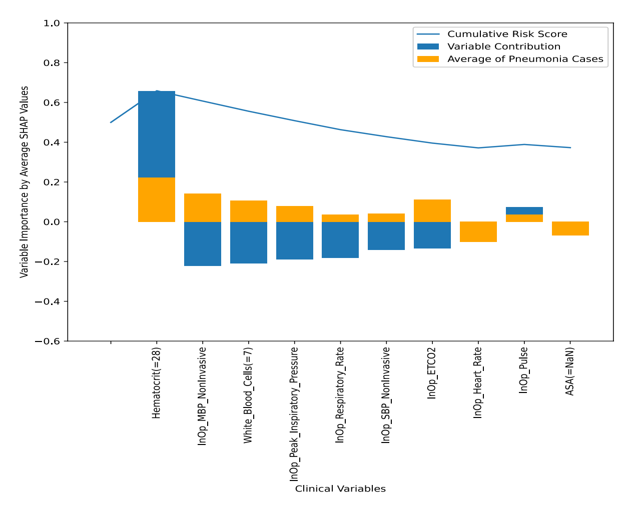

a

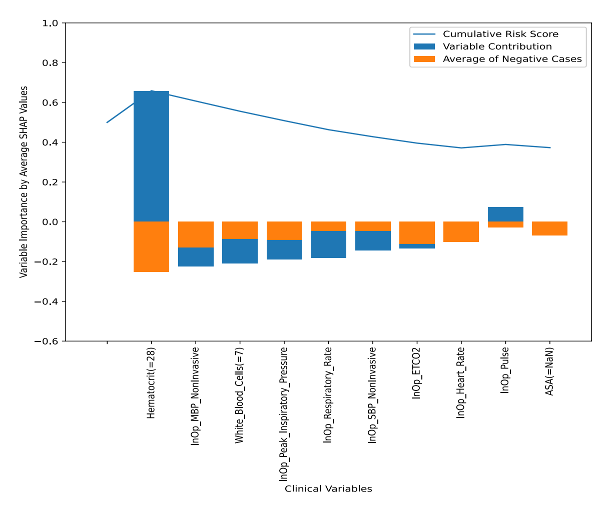

b

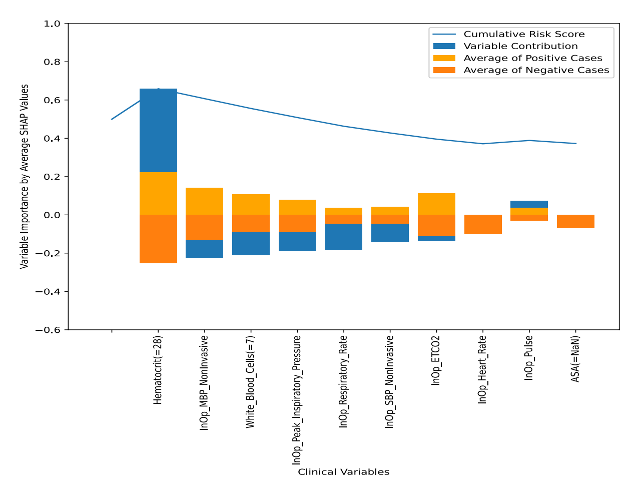

c

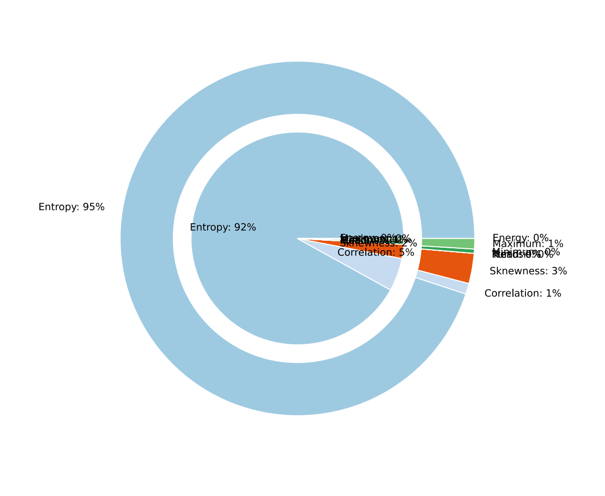

d

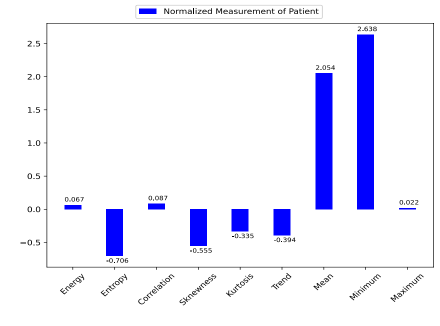

e

eFigure 2: Patient 2 - negative prediction of pneumonia

© 2021 Xue B et al. *JAMA Network Open.*

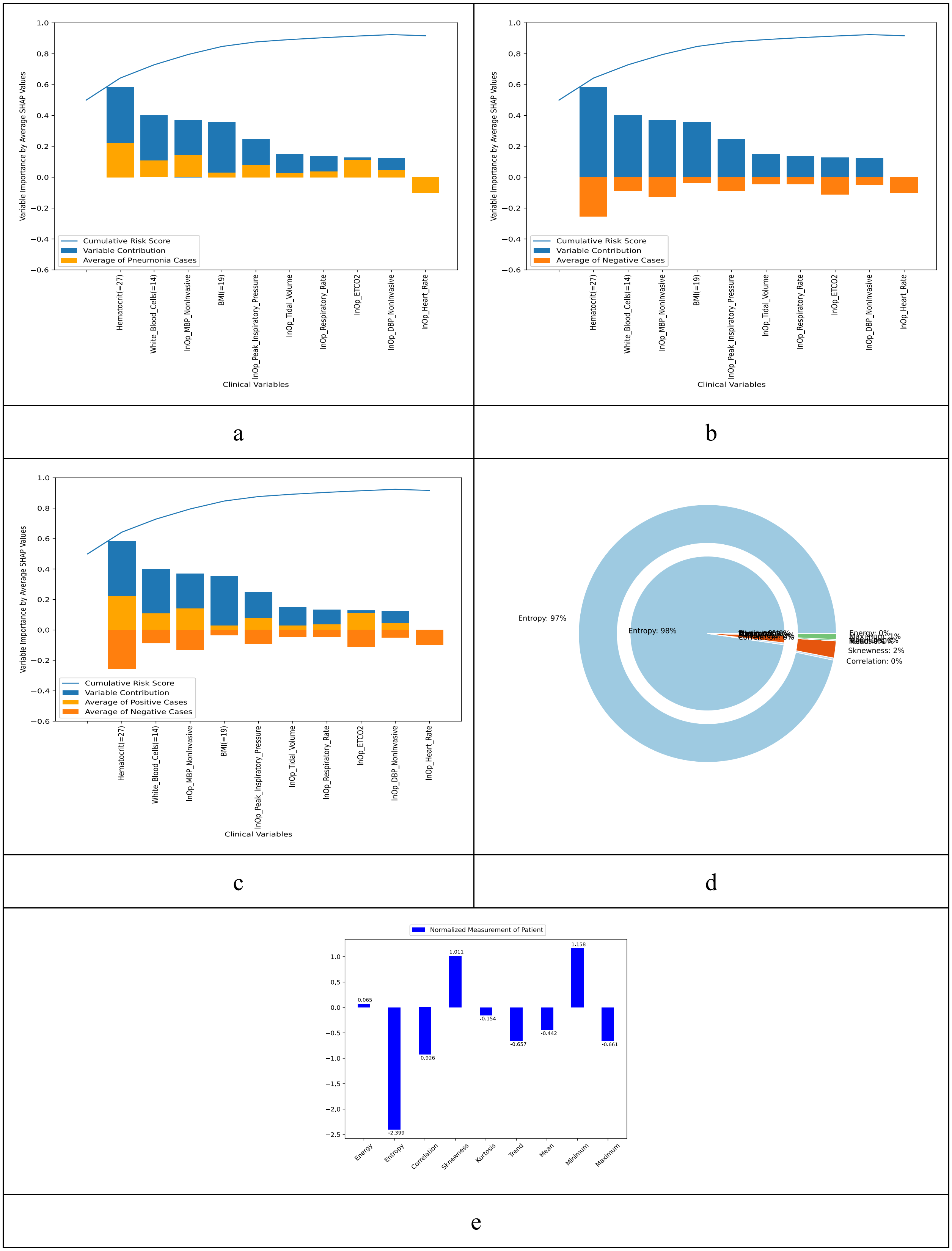

eFigure 3: Patient 3 - positive prediction of pneumonia

© 2021 Xue B et al. *JAMA Network Open.*

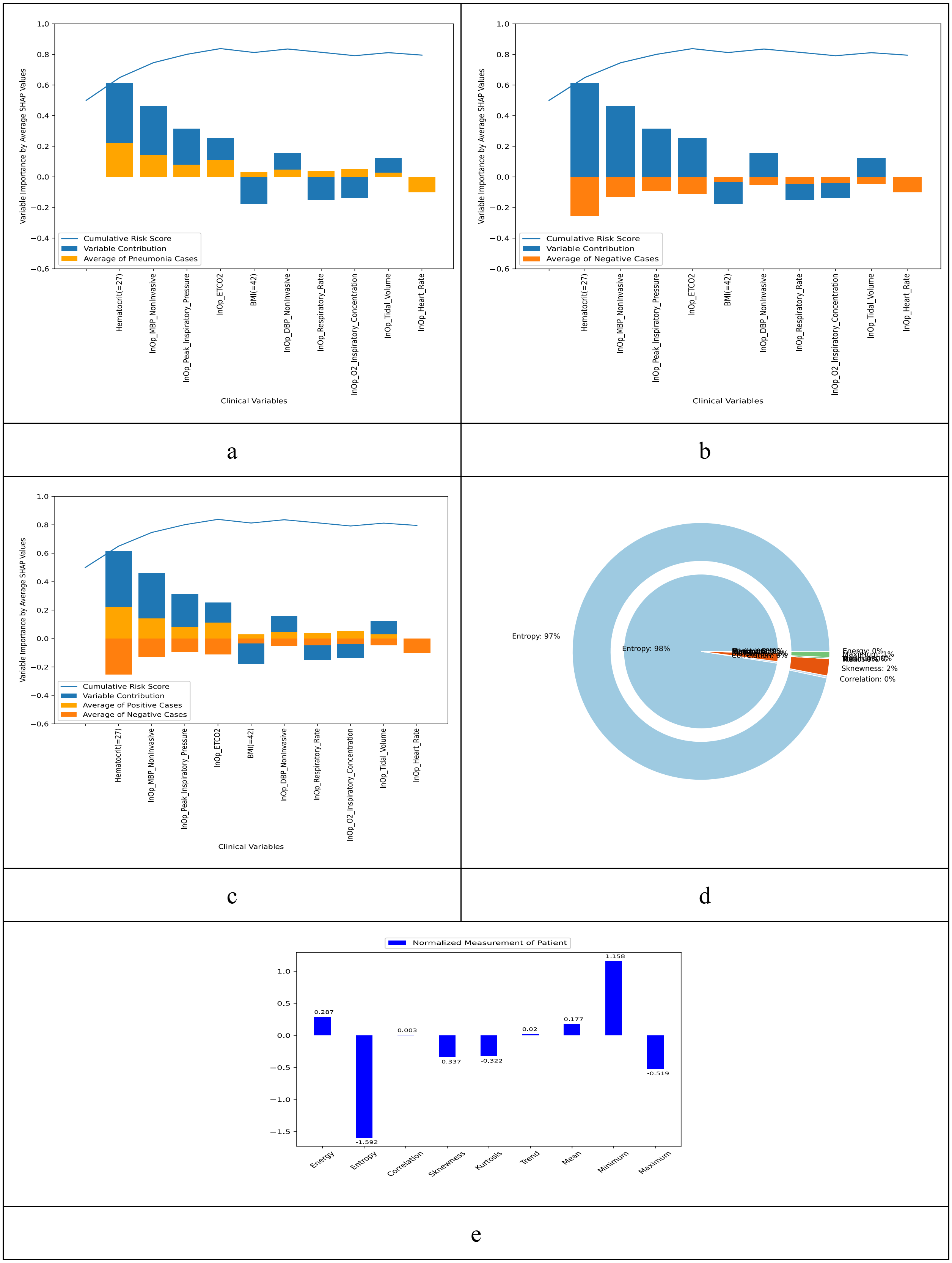

eFigure 4: Patient 4- positive prediction of pneumonia

© 2021 Xue B et al. *JAMA Network Open.*

eAppendix 7. Model Interpretation on False Positives, False Negatives, True Positives, and True Negatives

In this section, we show example cases of true positives (TP), false positives (FP), true negatives (TN) and false negatives (FN) for 5 outcomes based on model interpretation. For the 20 example cases, we show: the comparison of patients with respect to the risks of getting pneumonia (TP: Fig 6.a, FP: Fig 6.b, TN: Fig 6.c, and FN: Fig 6.d); the comparison of the patient with respect to the risks of getting AKI (TP: Fig 7.a, FP: Fig 7.b, TN: Fig 7.c, and FN: Fig 7.d); the comparison of patients with respect to the risks of getting DVT (TP: Fig 8.a, FP: Fig 8.b, TN: Fig 8.c, and FN: Fig 8.d); the comparison of patients with respect to the risks of getting PE (TP: Fig 9.a, FP: Fig 9.b, TN: Fig 9.c, and FN: Fig 9.d) and the comparison of patients with respect to the risks of getting delirium (TP: Fig 10.a, FP: Fig 10.b, TN: Fig 10.c, and FN: Fig 10.d).

In each figure, we are showing 4 distinct cases in the prediction of same outcome. In subplots a and c, most of the patients' preoperative and intraoperative data were consistent with historical cases of positive/negative patients, which resulted in constantly increasing or decreasing risks. In subplots b and d, the patients' data had mixed effects: some measurements were consistent with positive cases but some measurements were consistent with negative cases, hence the overall risk fluctuated. Taking Fig. 6 for example, some measurements including albumin, hematocrit were weighted as more important in the predictive model of pneumonia, thus the values of these measurements misled the overall risk. Note that albumin level in the case of Fig. 6c and 6d were missing. In the presence of missingness, machine learning model learned from historical data that indication of missing measurement would lower the likelihood of getting pneumonia, however, this was not always true and such missingness misled the prediction in Fig. 6d.

By looking at different scenarios in each outcome, we argue that the model interpretation have several advantages than a simple risk score. First, regardless of the correctness of the prediction, model interpretation could provide us with the important variables in each case, and how such variables affect the prediction risk in comparison with the positive/negative cohorts. Second, when most measurements are consistently contributing towards the same direction, such model interpretation could provide clinicians with more confidence in trusting the prediction risks. Last but not least, outcomes and input variables may not have a simple causal relationship. In the cases of false negatives and false positives, the conflicting factors could highlight the issues of wrong data, or alert clinicians the complexity of the patients' scenario, so that more attention should be paid to the details.

© 2021 Xue B et al. *JAMA Network Open.*

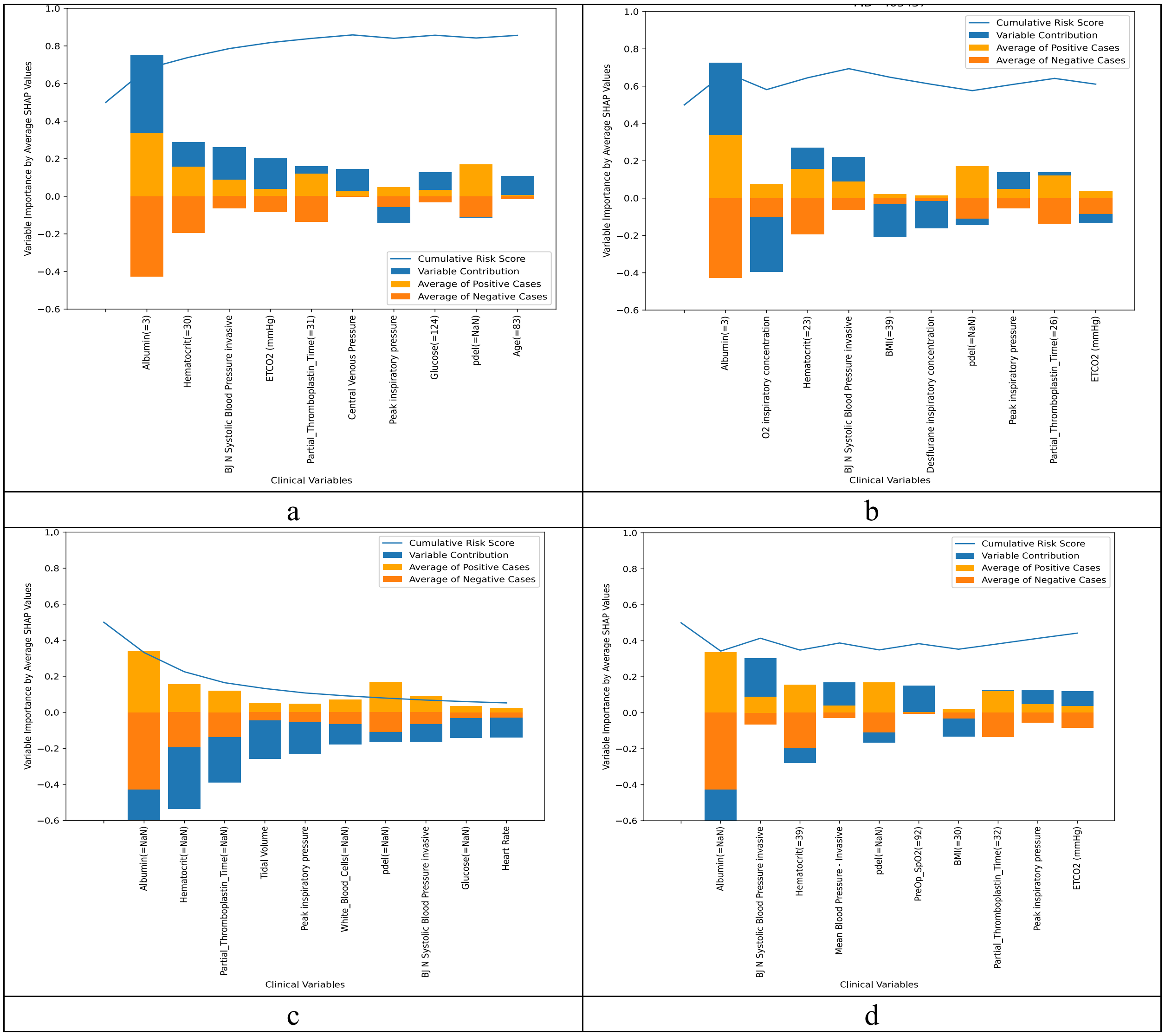

eFigure5: Example cases of true positives (TP), false positives (FP), true negatives (TN) and false negatives (FN) for getting pneumonia

© 2021 Xue B et al. *JAMA Network Open.*

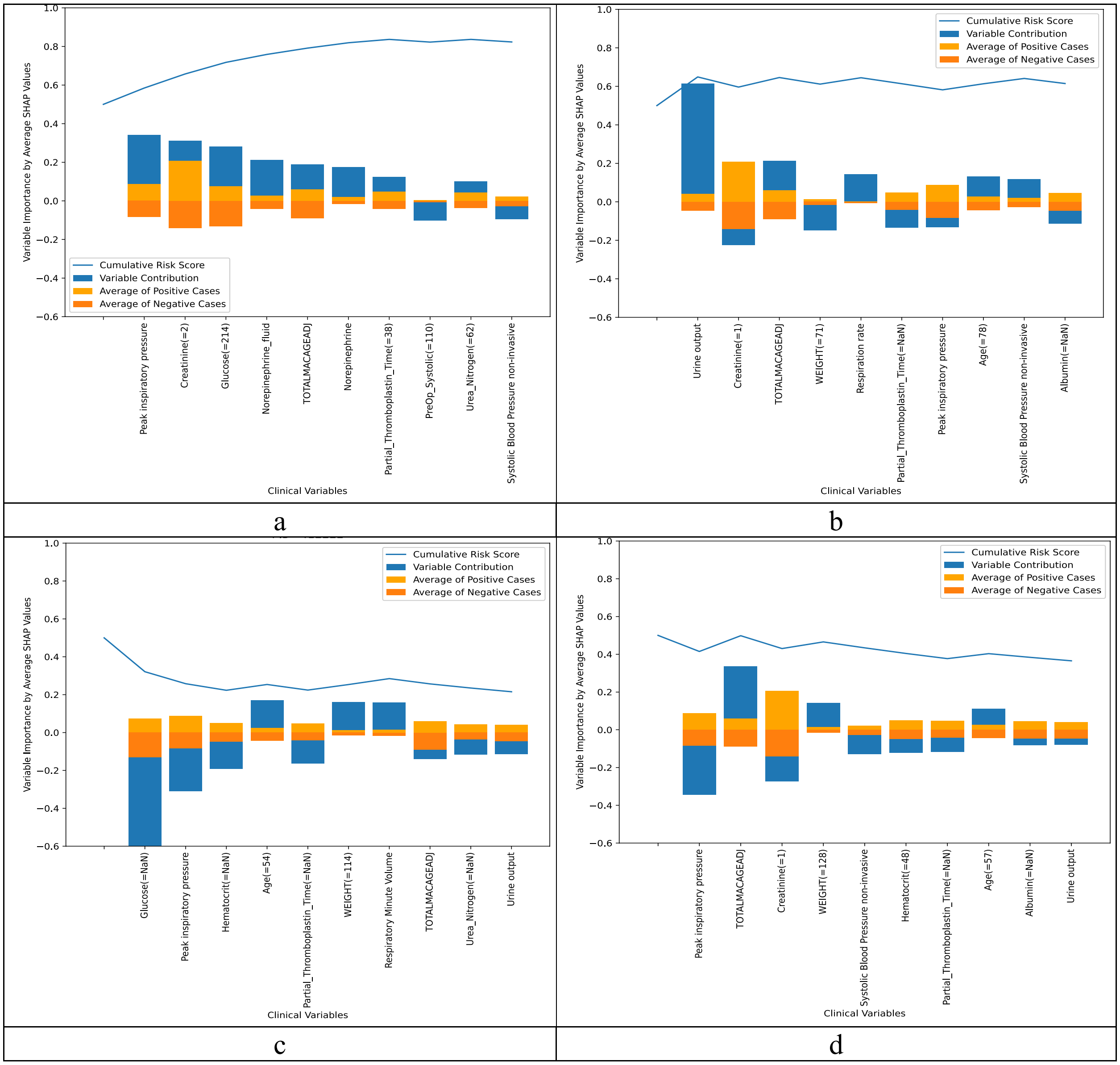

eFigure6: Example cases of true positives (TP), false positives (FP), true negatives (TN) and false negatives (FN) for getting AKI

© 2021 Xue B et al. *JAMA Network Open.*

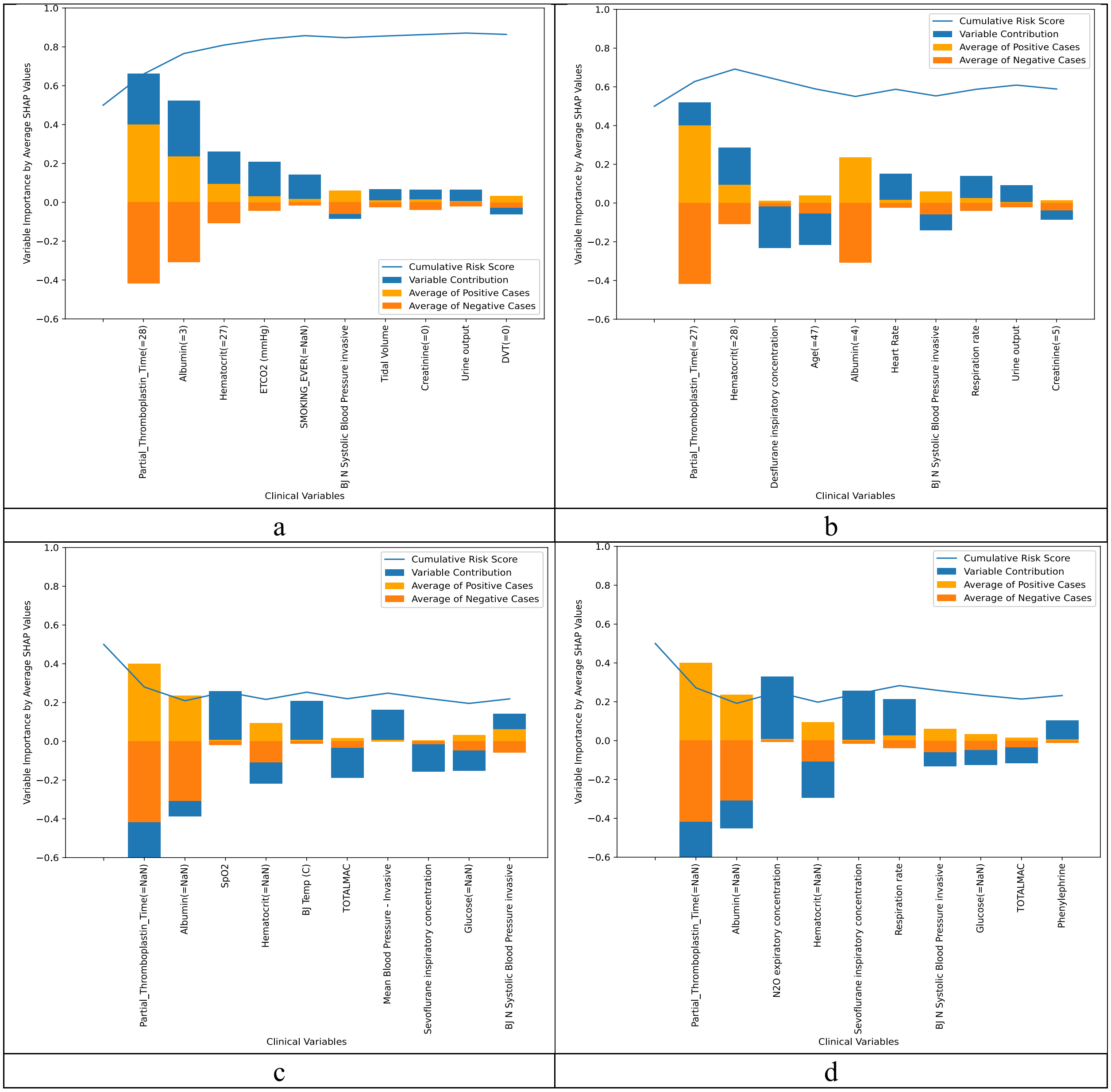

eFigure7: Example cases of true positives (TP), false positives (FP), true negatives (TN) and false negatives (FN) for getting DVT

© 2021 Xue B et al. *JAMA Network Open.*

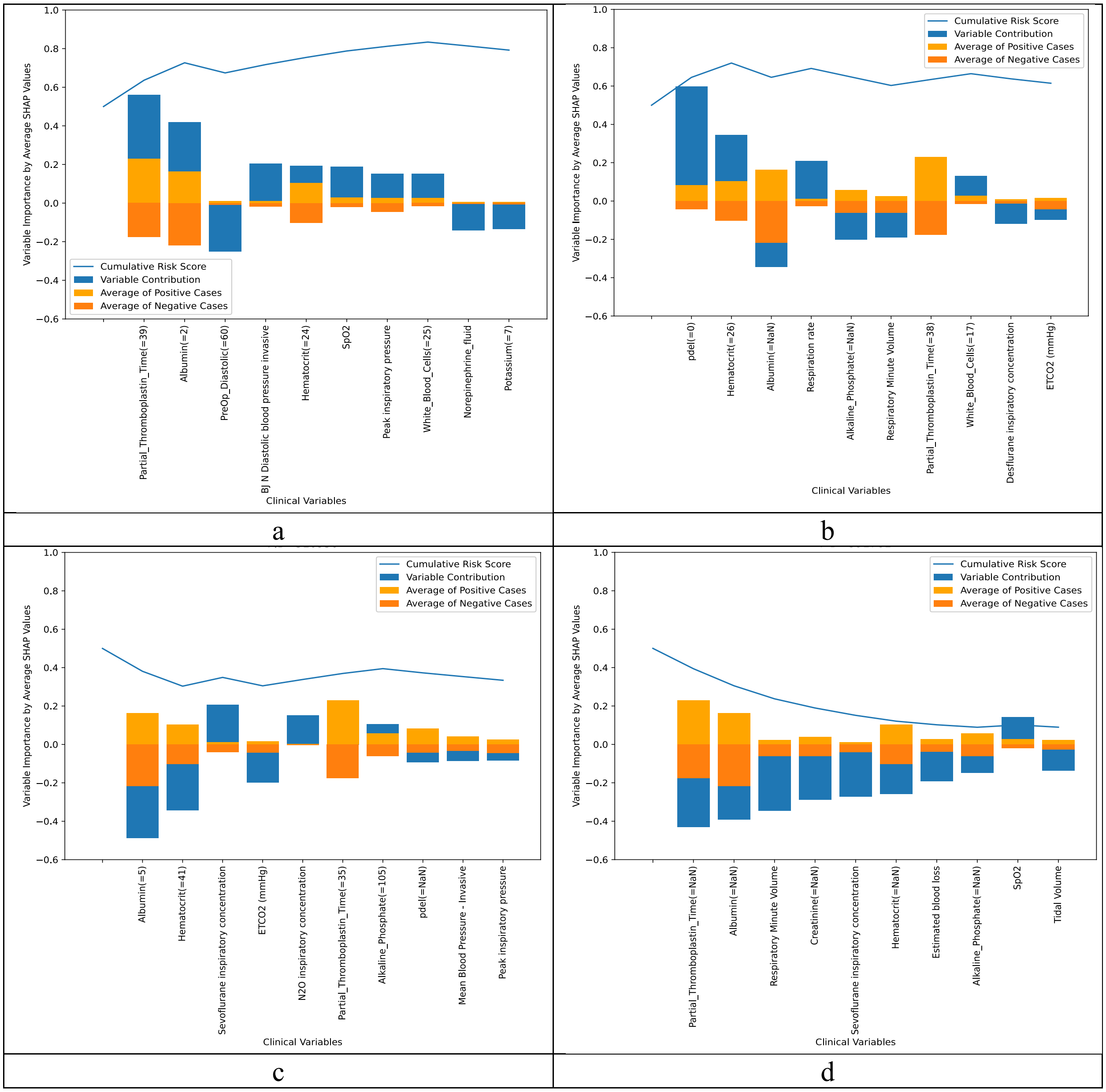

eFigure8: Example cases of true positives (TP), false positives (FP), true negatives (TN) and false negatives (FN) for getting PE

© 2021 Xue B et al. *JAMA Network Open.*

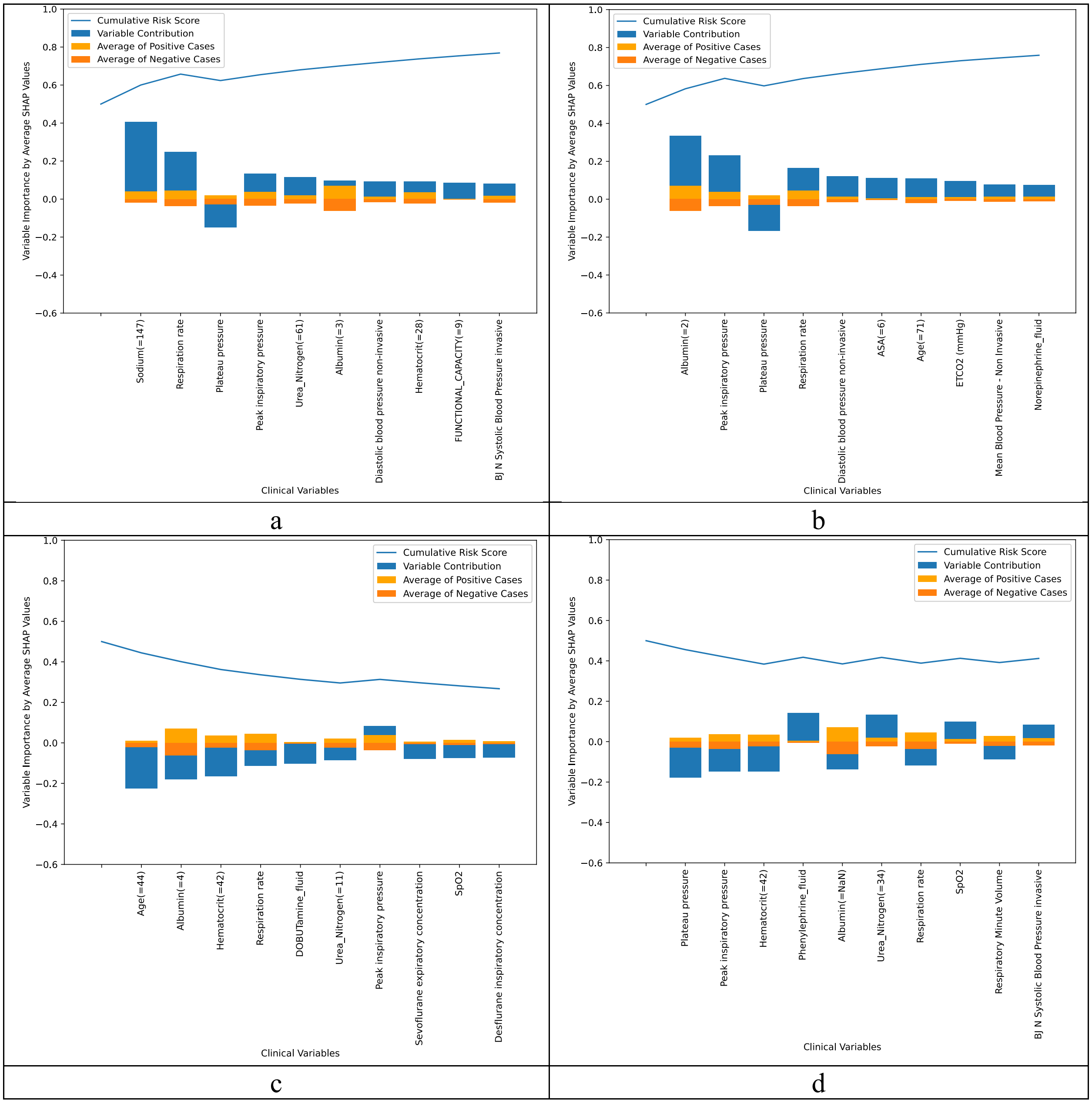

eFigure9: Example cases of true positives (TP), false positives (FP), true negatives (TN) and false negatives (FN) for getting delirium

© 2021 Xue B et al. *JAMA Network Open.*

eAppendix 8. Three Scenarios for Predicting Postoperative Complications

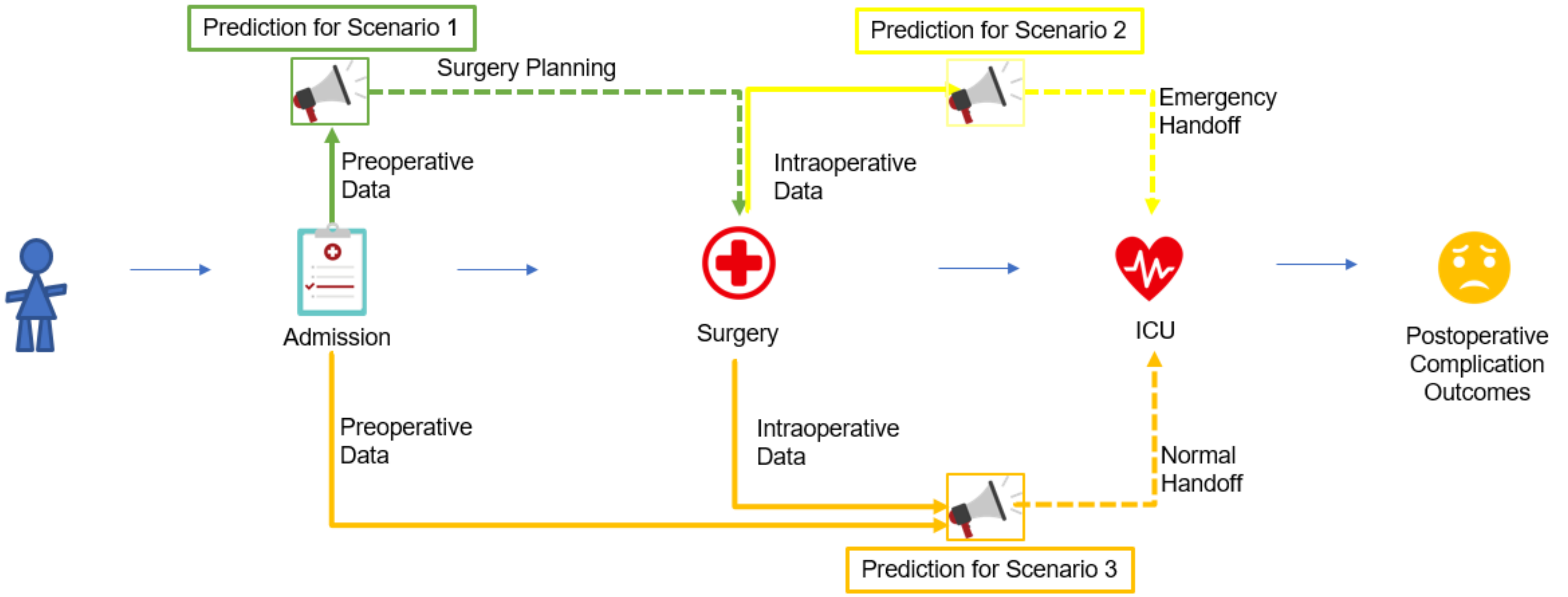

eFigure10: Three scenarios for predicting postoperative complications. Scenario 1 occurs at the admission stage and only preoperative data is used for surgery planning. Scenario 2 occurs after emergency surgery is performed and only intraoperative data is used for handoff process. Scenario 3 occurs after normal surgery with both preoperative and intraoperative data used for handoff process

© 2021 Xue B et al. *JAMA Network Open*.

References:

1. Davis J, Goadrich M. The relationship between Precision-Recall and ROC curves. In: *Proceedings of the 23rd International Conference on Machine Learning*. ICML ’06. Association for Computing Machinery; 2006:233–240. doi:10.1145/1143844.1143874

2. Azur MJ, Stuart EA, Frangakis C, Leaf PJ. Multiple imputation by chained equations: what is it and how does it work? *Int J Methods Psychiatr Res*. 2011;20(1):40-49. doi:10.1002/mpr.329

3. Stekhoven DJ, Bühlmann P. MissForest—non-parametric missing value imputation for mixed-type data. *Bioinformatics*. 2012;28(1):112-118. doi:10.1093/bioinformatics/btr597

© 2021 Xue B et al. *JAMA Network Open.*
